# Bumble bee diet breadth increases with local abundance and phenophase duration, not intraspecific variation in body size

**DOI:** 10.1007/s00442-024-05560-9

**Published:** 2024-05-25

**Authors:** Will R. Glenny, Justin B. Runyon, Laura A. Burkle

**Affiliations:** 1 Department of Ecology, Montana State University, Bozeman, MT USA; 2 US Department of Agriculture Forest Service, Rocky Mountain Research Station, Bozeman, MT USA

**Keywords:** Pollinators, Foraging, Networks, Species interactions, Species traits

## Abstract

Patterns of abundance across space and time, and intraspecific variation in body size, are two species attributes known to influence diet breadth and the structure of interaction networks. Yet, the relative influence of these attributes on diet breadth is often assumed to be equal among taxonomic groups, and the relationship between intraspecific variation in body size on interaction patterns is frequently neglected. We observed bee–flower interactions in multiple locations across Montana, USA, for two growing seasons and measured spatial and temporal patterns of abundance, along with interspecific and intraspecific variation in body size for prevalent species. We predicted that the association between spatial and temporal patterns of abundance and intraspecific variation in body size, and diet breadth, would be stronger for bumble bee compared to non-bumble bee species, because species with flexible diets and long activity periods can interact with more food items. Bumble bees had higher local abundance, occurred in many local communities, more intraspecific variation in body size, and longer phenophases compared to non-bumble bee species, but only local abundance and phenophase duration had a stronger positive association with the diet breadth of bumble bee compared to non-bumble bee species. Communities with a higher proportion of bumble bees also had higher intraspecific variation in body size at the network-level, and network-level intraspecific variation in body size was positively correlated with diet generalization. Our findings highlight that the association between species attributes and diet breadth changes depending on the taxonomic group, with implications for the structure of interaction networks.

**Supplementary Information:**

The online version contains supplementary material available at 10.1007/s00442-024-05560-9.

## Introduction

Species attributes related to interaction patterns are important to understand processes leading to the assembly of ecological networks (McGill et al. 2006). For an interaction between members of different trophic levels to take place, individuals must overlap in space and time at a sufficient abundance to produce random encounters (abundance) and have morphological features to exploit the resources provided by a partner (morphological matching) (Peralta et al. 2020b; Vazquez et al. 2009). Species attributes, such as variation in abundance and morphology, are, therefore, likely associated with the range of resources utilized by a species within a community (diet breadth) (Blüthgen et al. 2008). The association between species attributes and diet breadth may clarify how species affect the existence of competitors, flow of energy through trophic levels, respond to environmental change, and ultimately structure ecological interaction networks (Forister et al. 2015). However, the strength of association between species attributes and interaction patterns is often assumed to be consistent across groups of species with diverse life histories, when interaction patterns are likely the result of co-occurring species with diet breadths that are more strongly associated with patterns of abundance or morphological matching (Coux et al. 2021; Peralta et al. 2020a). Understanding the strength of association between abundance and morphological characteristics with the diet breadth of organisms may, therefore, be important to identify processes structuring interaction networks.

For bee species, the abundance across space and time and the morphological traits of individuals are attributes that contribute to diet breadth. Bee species that are locally abundant (Burkle et al. 2021; Chacoff et al. 2018; Resasco et al. 2021; Vazquez et al. 2009), occur in many local communities (hereafter, “common” species) (Wood et al. 2016), or have long phenophases (i.e., have an activity period that spans an extended portion of the growing season) (Burkle et al. 2021; Sponsler et al. 2022a) can have broad diets by overlapping with different flower species that occur across diverse habitats and periods of the growing season. Additionally, bee body size is a morphological trait that is related to metabolic demands (Heinrich 1975; Woodard and Jha 2017) and foraging distance (Greenleaf et al. 2007), and is allometrically related to tongue length, which influences the size and shape of flowers from which a bee can collect resources (Cariveau et al 2016; Kendall et al. 2019; Klumpers et al. 2019; Stang et al. 2009). The relationship between body size and diet breadth for bees can be positive (Smith et al. 2019) or negative (Raiol et al. 2021; Villalobos et al. 2019), depending on the ecosystem. However, the diet breadth of some species may be constrained by physiological requirements (Cane 2016; McAulay et al. 2021; Roulston and Goodell 2011), inherent preferences for specific flowers (Junker and Parachnowitsch 2015), and morphological adaptations that increase interaction efficiency (i.e., trait matching) (Klumpers et al. 2019; Stang et al. 2009). Consequently, the abundance of species across space and time and body size may have different strengths of associations with diet breadth, depending on the specific ecological requirements of groups of bee species.

Bumble bees (*Bombus* spp.) are ecologically unique within bee communities as large-bodied, eusocial diet generalists (Michener 2007; Woodard 2017). These attributes may increase the diet breadth of bumble bee species compared to other co-occurring solitary bee species. Within temperate ecosystems, bumble bees persist at high abundance across space and time, potentially as a result of physiological adaptations to tolerate cold and seasonal conditions (Woodard 2017). Bumble bee species that are widespread across time and space may, therefore, have broad diets if they are also flexible to interact with different plant species (Morán-López. et al. 2022). Additionally, high levels of variation in body size as a result of colony behaviors (Chole et al. 2019; Couvillon and Dornhaus 2009; Couvillon et al. 2010; Goulson 2010) can lead to a positive association with diet breadth (Peat et al. 2005) and population growth of some species (Austin and Dunlap 2019). By contrast, for most solitary bee species, which represent the majority of bee species within temperate ecosystems, a single individual provisions resources without cooperation from conspecifics for brood that emerge the following growing season (Danforth et al. 2019). Foraging individuals of solitary bees may, therefore, be constrained to collect resources from flower species that are active during a narrow range of time and space to provide specific resources required by developing larvae (Ogilvie and Forrest 2017; Roulston and Goodell 2011). Non-bumble bee species that are limited across time and space may require unique morphological characteristics to maintain consistent interactions with a narrow range of floral resources (Zhao et al. 2022). Comparing the strength of associations between abundance and body size, and diet breadth for bumble bee and non-bumble bee species, may help elucidate the drivers of diet generalization among species in ecological communities.

The association between body size and diet breadth may also depend on the composition and trait values of co-occurring bee species within local communities. The diet breadth of co-occurring pollinators can increase with co-occurring bumble bee abundance (Fontaine et al. 2008; Hallett et al. 2017) and floral fidelity may decrease when co-occurring bumble bees are removed (Brosi and Briggs 2013). Individuals of bumble bee species potentially occupy a similar trait space and could be prone to competitive exclusion in some ecosystems (Inouye 1978; Pyke 1982; MacArthur and Levins 1967). Local communities that have a high abundance of closely related species may exhibit a tighter trait space and higher levels of competition for resources (Grime 2006; HilleRisLambers et al. 2012). Simultaneously, species with high intraspecific variation in body size may occupy a wider dietary niche and could avoid competition with closely related species by increasing interaction flexibility (Bolnick et al. 2011). High levels of intraspecific body size variation in bumble bee species may confer foraging flexibility and diet expansion when conspecific densities are high (Fontaine et al. 2008). Intraspecific variation in body size, therefore, is a necessary, and often overlooked, measurement required to understand the diet breadth of species in ecological communities (Violle et al. 2012). Local assemblages with high relative abundance of bumble bees may have low interspecific variation in body size and high intraspecific variation in body size at the community level, leading to overall high levels of diet generalization in bee–flower networks.

**Fig. 1:**
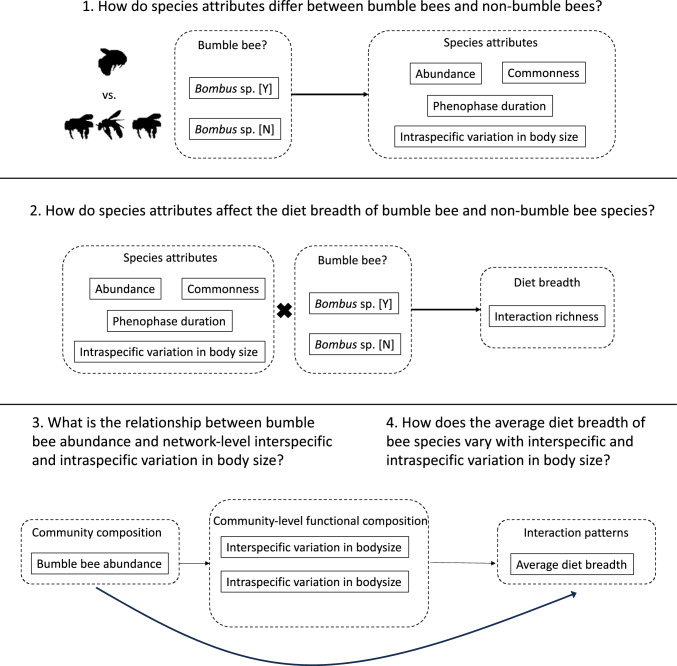
Hypotheses tested within this study. Each section depicts the model structure used to test for an association between, (1) Status as a bumble bee and species attributes associated with abundance across space and time, and intraspecific variation in body size; (2) The interactive effect between these species’ attributes and status as a bumble bee, and diet breadth; and (3) How bumble bee abundance may have a direct effect on community-level inter- and intra-specific variation in body size and; (4) the subsequent effects on community interaction patterns

We observed bee–flower interactions from six locations across two years in Montana, USA, and measured the body size and abundance of all female individuals caught to understand how species attributes and community composition influence interaction patterns across groups of bee species. Among bee species that are prevalent within this system, we compared the strength of association between local abundance, commonness, phenophase duration, and intraspecific variation in body size on diet breadth in *Bombus* and non-*Bombus* species. We asked, (1) how do species attributes (i.e., local abundance, commonness, phenophase duration, and intraspecific variation in body size) of bumble bee species compare to non-bumble bee species? We hypothesized that bumble bee species will be more locally abundant, common, have longer phenophases, and higher variation in body size compared to non-bumble bee species (Fig. 1). Additionally, (2) do attributes of bee species (i.e., local abundance, commonness, phenophase duration, and intraspecific body size variation) have a stronger association with diet breadth for bumble bee or for non-bumble bee species (Fig. 1)? We hypothesized that the diet breadth of bumble bee species will have a stronger association with patterns of abundance across space and time, and intraspecific variation in body size, compared to non-bumble bee species. Lastly, incorporating the network-weighted mean body size of all bee species and bee–flower interaction networks within a location and year we asked, (3) what is the relationship between bumble bee abundance, and the network-level interspecific and intraspecific variation in body size, and (4) what is the relationship between body size variation and average diet breadth across networks (Fig. 1)? We hypothesized that the relative abundance of bumble bees would have a negative association with the interspecific, but a positive association with the intraspecific variation in body size at the community level. Bumble bees may have greater intraspecific variation in body size than non-bumble bees and increase foraging flexibility and diet generalization when present at high densities in local communities.

## Materials and methods

### Site selection

**Fig. 2:**
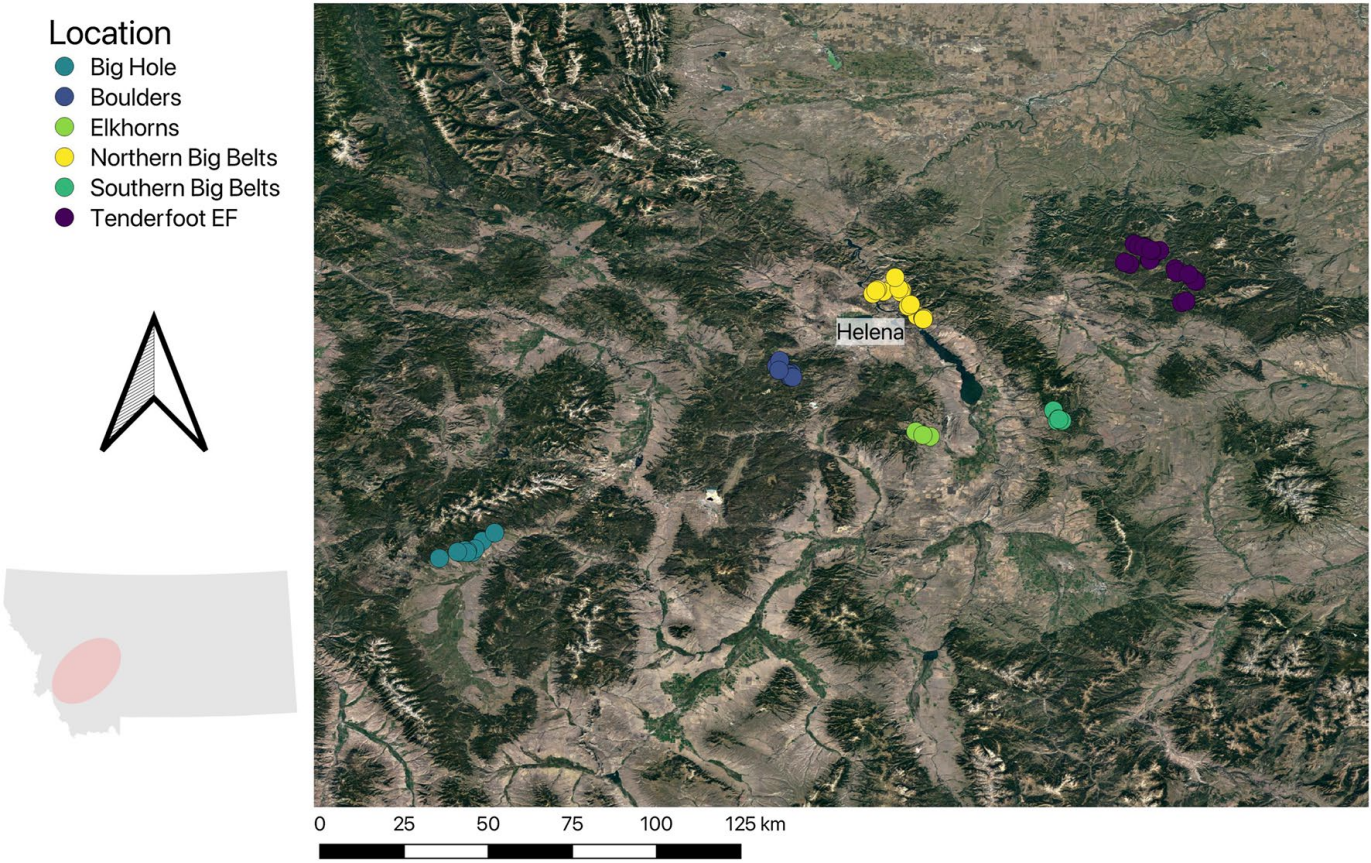
Map of the study area. Sites (circles) were distributed throughout locations (colors) to sample bee–flower interactions across the full range of environmental conditions in a location. The inset map illustrates the spatial extent (pink oval) of the locations within west-central and southwest Montana (color figure online)

We quantified bee–flower interactions from six locations in west-central and southwest Montana U.S. The locations were distributed across west-central Montana in semi-natural ecosystems and are characteristic of habitats that are common to the intermountain west (Fig. 2). The Northern Big Belts are conifer-grasslands dominated by ponderosa pine (*Pinus ponderosa*), Southern Big Belts are post-disturbance lodgepole pine forests (*Pinus contorta*), Elkhorns are sagebrush steppe (*Artemisia* spp.), Boulders are mixed-conifer forests, Tenderfoot Experimental Forest (Tenderfoot EF) is montane grasslands, and the Big Hole is a sagebrush-forest ecotone (Supplemental Table 1). The proportion of individuals caught from each location that were bumble bees ranged from 8.7 to 61.4% (Supplemental Table 1).

Multiple sites within each location were established to observe bee–flower interactions across the range of ecological conditions that exist within each location. In the locations around Helena, sites were established depending on the presence and abundance of focal plant species (Supplemental Table 2). Big Hole sites were distributed along the boundary between shrubland and forest habitat types. Twelve Big Hole sites were randomly placed at locations that (a.) contained both shrub and tree species, (b.) tree canopy cover was < 10%, (c.) slope < 30%, and were (d.) separated by at least 110 m (details included in (Glenny et al. 2023). As a result of these two distinct site selection strategies, the number of sites, minimum distance between sites and total area sampled differed by location. Within a location, the number of sites ranged from 3 to 21 (median number of sites across all locations = 10.5), sites were separated by at least 217–2209 m (median minimum pairwise distance between sites within a location = 280 m), and sites were separated by at most 4406–28,064 m (median pairwise distance between sites within a location = 13,294 m) (Supplemental Table 1).

### Bee–flower observations

To determine the diet breadth of bee species within this study, we conducted timed observations (observation periods) of bee–flower interactions at each site during the summers of 2018 and 2019. Observation periods at sites began when flowers emerged following snowmelt (between May and June) and ended when flowers produced seed in August. In both years, locations were visited multiple times throughout the growing season at semi-regular intervals which tracked the emergence of new flowering species and sampled members of the bee and flower communities that are active during different times of the growing season. Within each location, observation periods were typically conducted at a site once per day (average of five sites per day of observation), and all sites within a location were observed at least once over the course of a week. The flowering plant communities at some sites were a combination of shrub and herbaceous species and were based on the presence of focal plant species (Supplemental Table 2). For flowering shrubs that were tall, had large floral densities, and distributed at low densities (*Salix bebbiana* and *Cornus sericea*) pollinator visitation was quantified during independent observation periods on multiple individual plants at each site. Consequently, sites with shrub species had a higher number of observation periods when compared to sites without shrub species. Some sites were affected by grazing, wildfire, or road decommissioning, which reduced access to sites and the number of observation periods that were conducted between years. The number of observation periods of each site was variable and ranged from 1 to 52 (median = 5) across both years. To improve the consistency of sampling events at sites across all locations, we removed any sites that were only observed once during the study (*n* = 5).

During each observation period (60 min for locations around Helena, and 120 min total for sites in the Big Hole), two observers hand-netted bees that interacted with the reproductive parts of any flower. The identity of the flower species from which a specimen was collected and all co-occurring flowering species were recorded. Total floral abundance was estimated by summing the number of open inflorescences of all co-occurring flowering plant species. All observation periods were conducted during warm and calm days, under clear skies, during the peak pollinator activity period (0900–1700 h) (Gibson et al. 2011). For *Salix bebbiana* and *Cornus sericea*, observations were conducted on a randomly selected individual for 5–10 min (10–20 min total), and multiple individuals were observed within the same site until the total sampling time resembled other sites sampled on the same day. Bee specimens collected in the field were kept and identified in the laboratory to the lowest taxonomic level possible (species or morphospecies, using published keys for bees within the region; see (Reese et al. 2018)), and with the help of expert taxonomists. Specimens are deposited in the Montana State University Pollinator Health Center Collection.

### Measuring the body size of bee individuals

The allometric relationship between the intertegular distance (the width between the wing bases on the dorsal side of the thorax; ITD) and body size of a bee individual results in an easily measurable trait to understand how body size varies between and within bee species (Cariveau et al 2016; Kendall et al. 2019). We measured the ITD for each female bee specimen collected from sites included within the analysis (*n* = 6,684; individuals; *n* = 266 species) using a dissecting microscope and ocular micrometer (Leica, Wetzlar, Germany). Variation in ITD among all individuals of the same species within a location and year was used to estimate intraspecific variation in bee body size (details in analytical methods below).

### Aggregating bee–flower interactions

Bee–flower interactions were aggregated across all observation periods within a location and year (hereafter referred to as a ‘network’) to include the full range of bee–flower interactions and reliably estimate intraspecific variation in body size and diet breadth for multiple bee species. The aggregation of observation periods resulted in 12 total networks (*n* = 6 locations x *n* = 2 years), and the total observation time for a network ranged from 20 to 256 h (median observation time across networks = 65.55 h). This approach is warranted given that the broad spatial extent and heterogeneous growing seasons of locations in this study limited the frequency of observation periods to sites and reduced the temporal resolution of sampling events.

## Analytical methods

### How do species attributes differ between bumble bees and non-bumble bees?

To compare interaction patterns between species across networks of different sizes and sampling intensities, we generated a rarefied network from each observed network by randomly selecting without replacement 96 bee individuals, which is equivalent to the least sampled network in this study (Olito and Fox 2015). The ITD, site, co-occurring floral species richness and floral abundance, and date captured remained associated with each bee individual selected during rarefaction. Results from rarefaction were checked to ensure that the individuals drawn during rarefaction cover the full range of observation dates for each network (mean rarefied sampling coverage across networks = 65–100 percent; median = 0.97) (Supplemental Table 1). Local abundance was calculated for each species as the average number of individuals caught per site where a species was detected. Commonness was calculated for each species as the number of sites where a bee species was captured as a proportion of all the sites in the rarefied network. Phenophase duration was calculated for each species as the difference in days between the earliest and latest day when an individual of a species was caught as a proportion of the total days sampled in a rarefied network. Intraspecific variation in body size was calculated for each species as the coefficient of variation (CV) in ITD for all individuals of the same species in a rarefied network (details below; Supplemental Table 3) (Jung et al. 2010). Species with fewer than two individuals sampled as a result of rarefaction were excluded from each rarefied network because the CV could not be calculated. Co-occurring floral species richness and abundance was averaged across all individuals of the same species within a rarefaction run to estimate the abundance of co-occurring floral resources. This process was repeated 100 times for each network. Mean rarefied abundance per site, commonness, phenophase duration, and CV was then calculated for each bee species. As a result of rarefaction, the relative abundance of each bee species within a network was preserved because abundant species were more likely to be drawn during each sampling event and the variation of body size, commonness, and phenophase duration for each species were comparable across networks because bee communities were sampled with equal intensity.

To help normalize the distributions of explanatory and response variables and meet the assumptions for statistical analysis, we only included species that were sampled from more than one site and had a phenophase duration greater than one day. Our inferences, therefore, pertain to a subset of prevalent bee species within this system (*n* = 128 species) (Supplemental Table 4). All covariates were checked for multicollinearity and had correlation coefficients of < 0.36.

We used linear mixed effects models (LMEMs) to test the hypothesis that bumble bee species are more locally abundant, common, have longer phenophases, and higher variation in body size compared to non-bumble bee species. The mean rarefied abundance, commonness, phenophase duration and CV of body size was used as response variables to ensure characteristics were compared between species sampled with equal effort. We visually evaluated the distribution of all variables for normality using histograms. All variables except phenophase duration were log-transformed to ensure normality. We used a multivariate analysis of variance (MANOVA) to test for an effect of genus *Bombus* on the potentially correlated response variables of rarefied abundance, commonness, phenophase duration, and CV of body size. A significant MANOVA (Pillai = 0.12, F_4,307_ = 10.9, *p*-value < 0.001) was followed by four separate linear mixed effects models (LMEM) to test for the effect of status as a *Bombus* species on mean rarefied commonness, abundance, phenophase duration, and CV of body size compared to non-*Bombus* species. Within each model, status as *Bombus* vs. non-*Bombus* was a main effect, and location, year, and species were fit as separate crossed random effects to account for sampling the same species within multiple locations, and the same location across multiple years. Results from each model were summarized with F-tests using the Satterthwaite’s method to estimate denominator degrees of freedom in the lmerTest package (Kuznetsova et al. 2017). Residual plots were used to check that the statistical assumptions were met for each model.

### How do species attributes affect the diet breadth of bumble bee and non-bumble bee species?

The mean rarefied interaction richness was used to describe the diet breadth of bee species, and was calculated as the total number of unique flower species visited by a bee species within a rarefied network, averaged across the 100 rarefied networks. Interaction richness is the raw number of partners observed for each bee species within a network and is complimentary to other species-level diet breadth metrics because it does not distinguish between strong or weak interactions, is not standardized against other co-occurring bee species, and emphasizes rare interactions, to describe the full range of potential food items that is visited by an individual bee species. We included bee species with a mean rarefied interaction richness of greater than one to meet the assumptions for statistical analysis. Mean rarefied interaction richness was log-transformed for normality.

We used an LMEM to test the hypothesis that high local abundance, commonness, long phenophase durations, and intraspecific variation in body size have a positive effect on the diet breadth of bee species. Within the model, mean rarefied interaction richness was the response variable, and mean rarefied local abundance, commonness, phenophase duration, and CV of body size were included as main covariates. After confirming that mean rarefied co-occurring floral richness and abundance were not correlated, we included both as covariates in the model to account for variation in diet breadth of each species that is explained by floral resources. We also included a categorical variable for the status of a bee species as a *Bombus* as a main effect to test the hypothesis that *Bombus* species had broader diet breadths when compared to non-bumble bee species, while controlling for differences in local abundance, commonness, phenophase duration, and CV of body size. Furthermore, to test the hypothesis that the strength of mechanisms related to diet breadth are different between groups of bee species, we also included an interaction between each species attribute and the status as *Bombus*. Location, year, and species were included as separate crossed random effects to account for sampling the same species in multiple locations and years. Results from this model were summarized using the F-tests as described previously.

### What is the relationship between bumble bee abundance and the network-level interspecific and intraspecific variation in body size?

We tested the relationship between bumble bee abundance and network-level interspecific and intraspecific variation in bee body size following the methods outlined by (Leps et al. 2006). A z-score transformation was applied to the ITD measurement of all individuals within the same observed network to interpret body size values on a similar scale. Using the z-score transformed estimate for body size, interspecific variation at the network-level was estimated as the difference in the mean value for a species compared to the average value for the community in a location and year, summed across all species in the community. To ensure that the average body size value for the network reflects the relative abundance of species within the network, we calculated the weighted mean body size for each species within a network as the mean body size for all individuals of a species scaled by the proportion within the network and summed across all species. For each species in a location and year, the difference between the average body size value and the weighted mean was squared and scaled by the proportion of the species within the network. Network-level interspecific trait variation was calculated by summing these values across all species at a location and year. Additionally, intraspecific trait variation for a network was calculated as the sum across species of the variance of the body size of all individuals of each species, scaled by the proportional abundance of the species within the network.

We then used two separate LMEMs to test for a relationship between bumble bee abundance and network-level interspecific and intraspecific variation in body size. Within each model, the proportion of all bees that were caught within a network that were individuals within the *Bombus* genus was used as a main effect, while location and year were separate random intercepts to account for repeated measures of bee communities within the same location across multiple years, and multiple locations within the same year. While bumble bee abundance is included in the response and predictor variables and may result in a correlation that reflects a mathematical consequence of shared data, the relationship between bumble bee abundance and network-level trait variation still allows us to understand how the composition of bees in a network alters trait overlap and variation among co-occurring species.

### How does the average diet breadth of bee species vary with interspecific and intraspecific variation in body size?

To understand the association between intraspecific and interspecific variation in bee body size and diet breadth among bees in a community, we calculated the average complementary specialization (d’; (Blüthgen et al. 2006) of bee species in an observed network.

d’ estimates the diet breadth of each bee species in a network based on the frequency of visitations observed to plant species, distinguishes between strong and weak interactions, and is relative to all other bee species present within the network. d’ is, thus, complementary to interaction richness as a measure of resource overlap and interaction partitioning between co-occurring bee species, and more appropriate to estimate the diet breadth of bee species within a network setting. Importantly, this measure of diet breadth incorporates a null model to ensure that diet breadth is not affected by network size and sampling intensity (Blüthgen et al. 2006). d’ is scaled between 0 (generalized—complete overlap in resources used) and 1 (specialized—no overlap in resources used) for each bee species and was averaged across all bee species in a network. We used the average d’, as opposed to H2’, because H2’ also considers the reciprocal specialization of flower species in the network-level metric and our study was only focused on bees (Blüthgen et al. 2006). We also calculated the average d’ based on the observed network (as opposed to a rarefied network) to use the complete assemblage of bee–flower interactions observed and ensure that the individuals sampled to determine diet breadth were consistent with the individuals sampled to calculate network-level inter- and intraspecific variation in body size (Supplemental Table 5).

We used a beta-regression mixed effects model (Cribari-Neto and Zeileis 2010) to test for a relationship between the average d’ and community-level inter- and intraspecific trait variation in body size. d’ is scaled between 0 and 1 and is, therefore, a proportional value that resembles a beta-distributed dependent variable. Within the model, we used the average d’ as the response variable, and the community-level measure of intra- and interspecific variation in body size as main effects, and location and year as separate random intercepts to account for locations that were resampled across multiple years.

### Statistical software used

All analyses were performed in the statistical software ‘R’ (R Core Team 2020). Network metrics were estimated using the ‘bipartite’ package (Dormann et al. 2008), LMEMs were constructed using the ‘lme4’ package (Bates et al. 2014). The beta-regression mixed-effects model was performed using the ‘glmmTMB’ package (Brooks et al. 2017).

## Results

### How do species attributes differ between bumble bees and non-bumble bees?

**Fig. 3:**
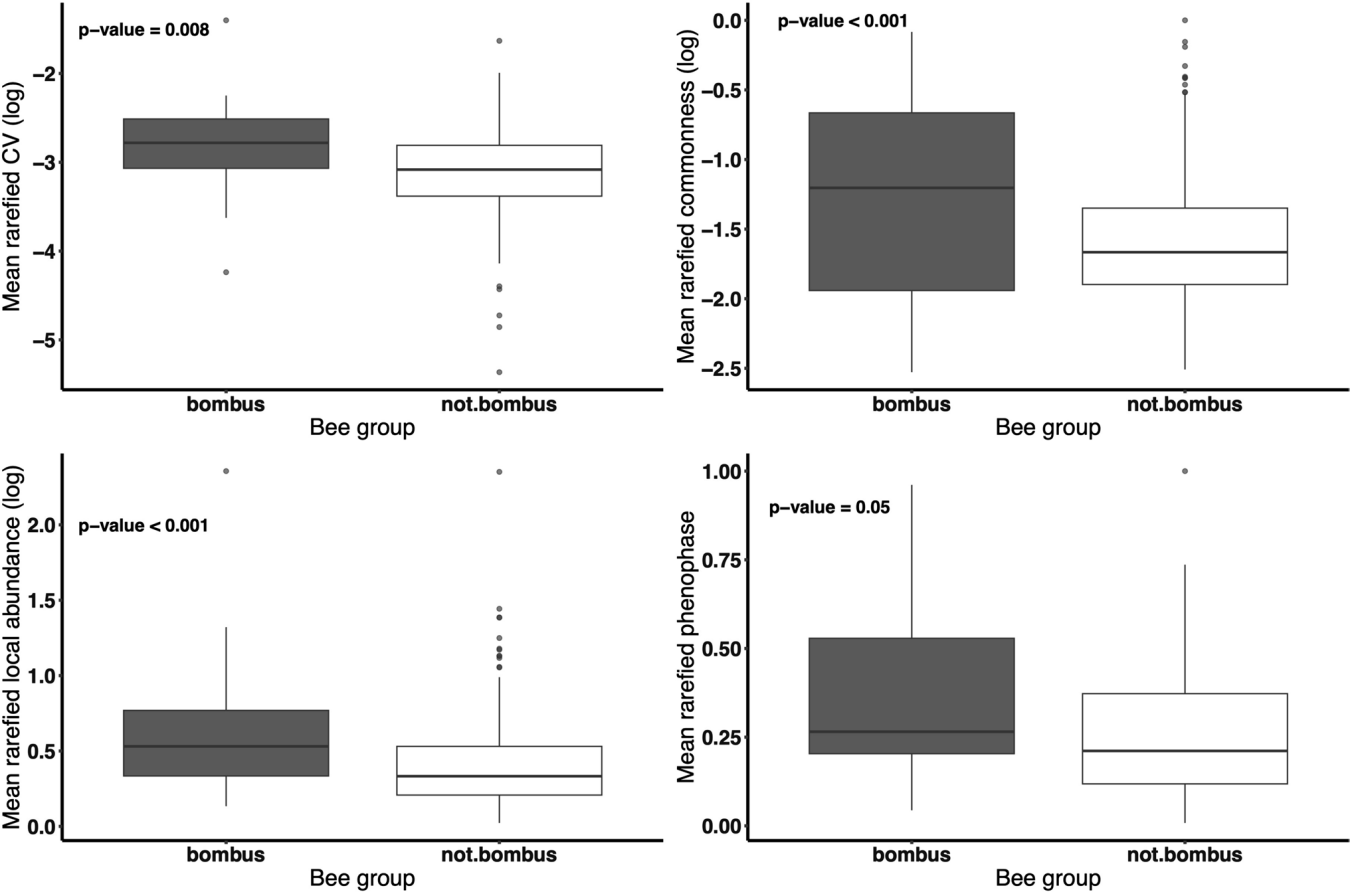
The mean rarefied coefficient of variation (CV) in body size (upper left), proportion of sites where a species was present (upper right) and local abundance (bottom left) was higher for *Bombus* species compared to non-*Bombus* species. All response variables except mean rarefied phenophase duration were log-transformed for normality. Error bars are ± one standard error. P-values correspond to the results from the linear mixed effects model reported in Table 1

Bumble bee species had higher levels of mean rarefied local abundance, commonness, phenophase duration, and variation in body size compared to non-bumble bee species (Fig. 3; Table 1).

**Table 1:** Summary of results from separate linear mixed effects models testing whether bumble bees differ from non-bumble bees in mean rarefied local abundance, commonness, phenophase duration, or intraspecific variation in body size

Response variable	Est	SE	Est. degrees freedom	*t*-value	*p*-value
Mean rarefied intraspecific variation in body size	0.26	0.09	46.78	2.65	**0.01**
Mean rarefied commonness	0.32	0.07	100.72	4.95	** < 0.001**
Mean rarefied local abundance	0.20	0.06	72.44	3.45	** < 0.001**
Mean rarefied phenophase duration	0.06	0.03	75.75	1.76	**0.05**

*P*-values are bolded when a significant (alpha < 0.05)

## How do species attributes affect the diet breadth of bumble bee and non-bumble bee species?

**Fig. 4:**
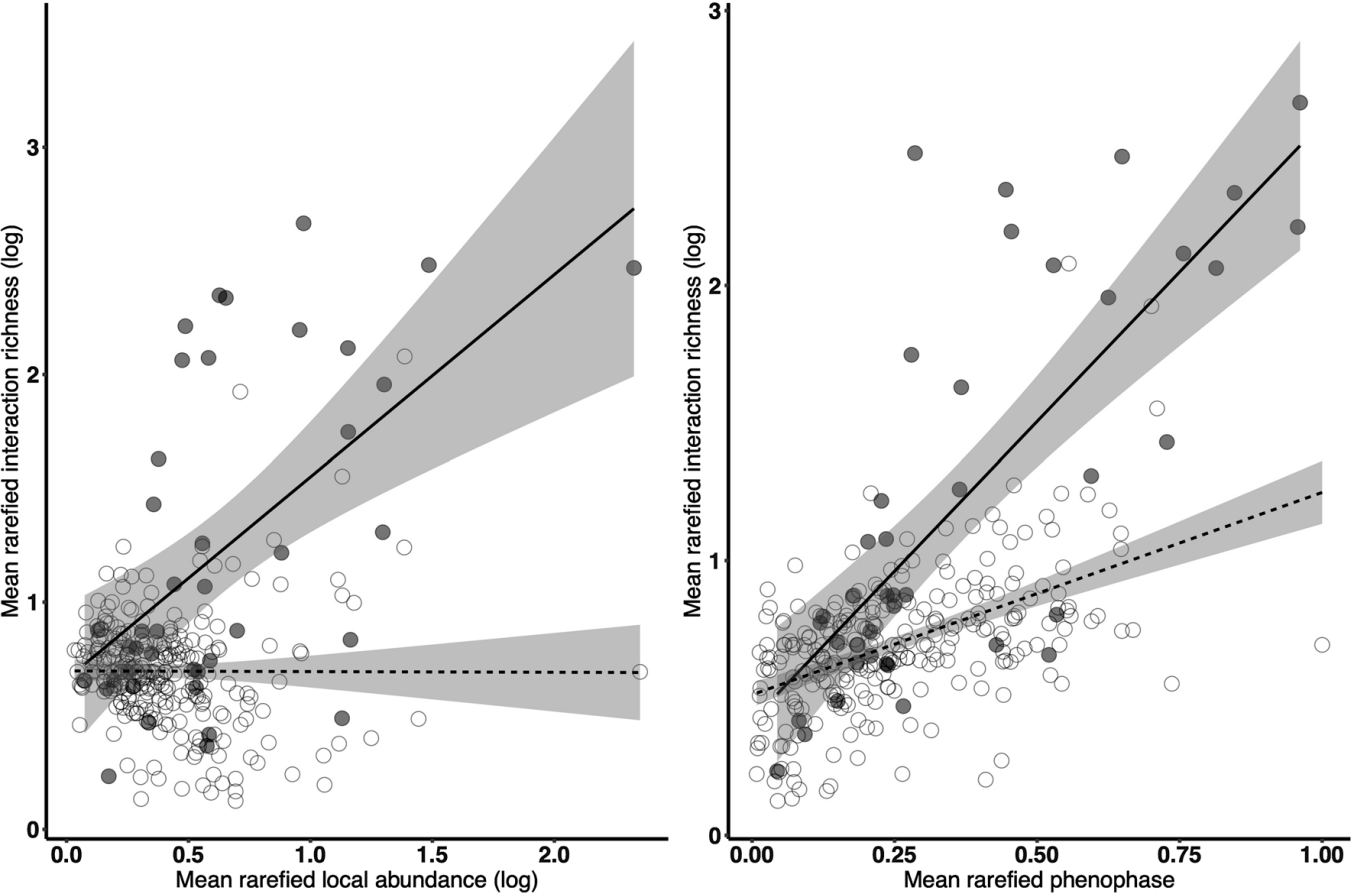
Mean rarefied interaction richness increased more positively for bumble bee species compared to non-bumble bee species with mean rarefied commonness (left) and phenophase duration (right). Points depict a bee species in a location and year. Bumble bee species are filled and non-bumble bee species are open circles. Error bars are ± *SE* and lines (solid for bumble bees and dashed for non-bumble bees) depict the best fit line with standard errors (grey shaded area). See Table 2 for complete model output

There was a positive association between local abundance, commonness, phenophase duration, and mean rarefied interaction richness across all bee species (Table 2). There was a stronger positive relationship of mean rarefied local abundance and phenophase duration on mean rarefied interaction richness for *Bombus* compared to non-*Bombus* species (Fig. 4; Table 2). After accounting for mean rarefied commonness, local abundance, phenophase duration, and floral resource availability, there were no main effects of status as a *Bombus* species or intraspecific variation in body size on mean rarefied interaction richness (Table 2).

**Table 2:** Summary of results from a linear mixed effects model testing for the effects of species attributes on diet breadth for bumble bee and non-bumble bee species. The ‘status as a *Bombus* species’, mean rarefied local abundance, commonness, phenophase duration, and intraspecific trait variation in body size were main effects along with separate two-way interaction terms with ‘status as a *Bombus* species’.

Covariate	Est	SE	Est. degrees freedom	*t*-value	*p*-value
Mean rarefied intraspecific variation in body size	0.15	0.51	294.29	0.29	0.76
Is *Bombus*	− 0.04	0.17	281.83	− 0.20	0.84
Mean rarefied floral richness	0.04	0.03	295.22	1.37	0.17
Mean rarefied floral abundance	− 0.001	0.009	289.76	− 0.17	0.86
Mean rarefied commonness	0.55	0.05	270.34	11.74	** < 0.001**
Mean rarefied local abundance	0.11	0.05	281.51	2.50	**0.01**
Mean rarefied phenophase duration	0.29	0.08	292.49	3.81	** < 0.001**
CV: *Bombus*	1.49	1.41	251.60	1.057	0.29
Mean rarefied commonness: *Bombus*	0.11	0.07	273.89	1.59	0.11
Mean rarefied local abundance: *Bombus*	0.21	0.10	255.89	2.18	**0.03**
Mean rarefied phenophase duration: *Bombus*	0.49	0.15	225.81	3.16	**0.001**

*P*-values are bolded when a significant (alpha < 0.05) correlation exists

### What is the relationship between bumble bee abundance and the network-level interspecific and intraspecific variation in body size?

**Fig. 5:**
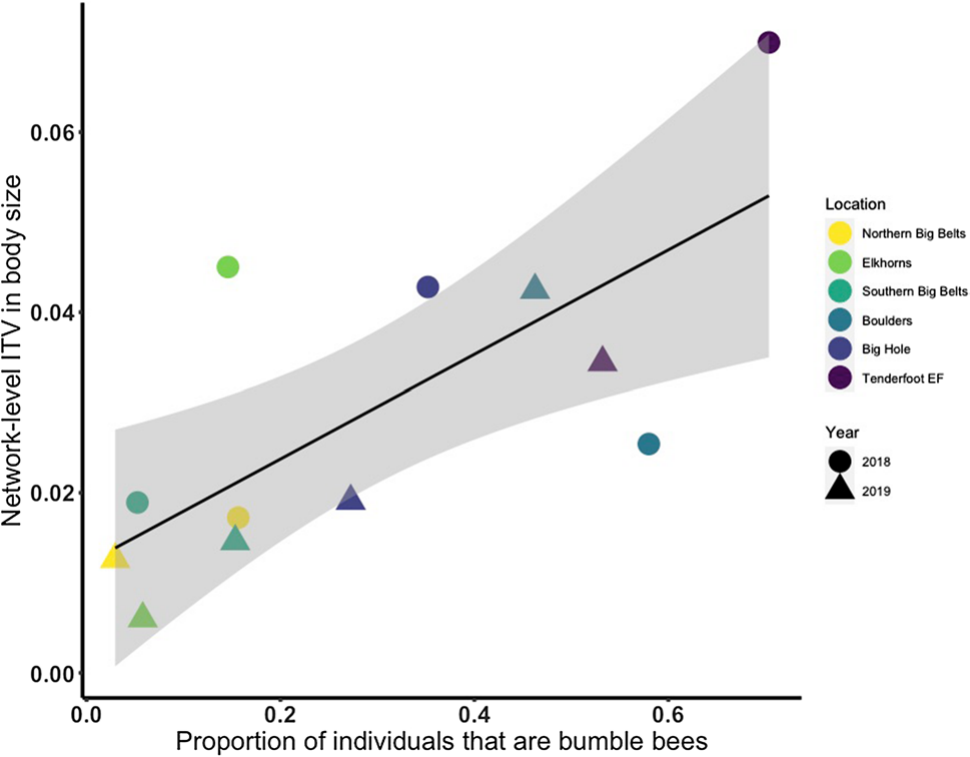
Network-level intraspecific variation (ITV) in body size increases with the proportion of individuals that are bumble bees. Each point is a network. The color of a point represents the location, and the shapes represent the year of sampling. The best fit line is surrounded by 95% confidence intervals (color figure online)

Total intraspecific variation in body size at the network-level increased with the proportion of bee individuals that are bumble bee species (Satterthwaite’s *t*-test, SE = 0.01, est.df = 9.27, *t*-value = 3.37, *p*-value = 0.007) (Fig. 5). We found no effect of bumble bee abundance on network-level interspecific variation in body size (Satterthwaite’s *t*-test, Est. = − 0.11, SE = 0.20, est.df = 4.47, t-value = − 0.53, *p*-value = 0.62).

### How does the average diet breadth of bee species vary with interspecific and intraspecific variation in body size?

We found evidence that network-level intraspecific variation in body size was negatively associated with the average diet breadth of bee communities (Beta-regression, estimate = − 5.77, SE = 2.96, *z*-value = − 1.95, *p*-value = 0.05). There was no relationship between network-level interspecific variation in body size and the average diet breadth of bee communities (Beta-regression, estimate = − 0.47, SE = 0.37, *z*-value = − 1.30, *p*-value = 0.20).

## Discussion

Body size and patterns of abundance are species attributes associated with the diet breadth of bees, but it remains unclear if the strength of these attributes on diet breadth are consistent across groups of species. We found that bumble bee species had higher levels of intraspecific variation in body size and were more locally abundant and common compared to non-bumble bee species, but only local abundance and phenophase duration had a stronger positive association with diet breadth for bumble bee species compared to non-bumble bee species. Bumble bee species often have large local populations that are active across the growing season, providing opportunities to interact with many flower species. There was no association between intraspecific variation in body size and diet breadth for bumble bee species. Strangely, while there was no association between intraspecific variation in body size and diet breadth for bumble bee species, communities with a higher proportion of bumble bee individuals had greater intraspecific variation in body size overall. In turn, there was a weak positive association between community-level intraspecific variation in body size and average diet breadth. The relative abundance of bumble bees may, therefore, be positively associated with the overall diet breadth of bee communities and potentially indicate that generalists have a strong effect on network structure. The strength of mechanisms that influence the diet breadth of bee species may be specific to groups of bees, which is important to understand processes leading to the assembly of interactions between species across trophic levels.

### How do species attributes differ between bumble bee and non-bumble bees?

Among bee species that are prevalent within this system, bumble bee species had higher levels of intraspecific variation in body size and were more common and abundant compared to other non-bumble bee species, which may be a function of bumble bees as large-bodied, eusocial species that are well-adapted to temperate ecosystems. We observed that bumble bees were most abundant within locations that were high elevation montane wetlands (Tenderfoot EF) and least abundant in locations at low elevations in dry shrub and grasslands (Northern Big Belts) (Supplemental Table 1). Eusocial species that produce colonies of individuals with a wider range of body sizes (Chole et al. 2019; Couvillon and Dornhaus 2009; Couvillon et al. 2010; Goulson 2010) may confer flexibility among populations to respond to variable environmental conditions found in temperate ecosystems. Bumble bee species with higher variation in body size may, therefore, have populations that can disperse to forage on patchily distributed floral resources (Warzecha et al. 2016) or remain active across a range of inclement conditions. Additionally, temperate ecosystems are areas that are cool and wet with a deeper snowpack and may have higher floral abundance when bumble bee colonies transition from a solitary to social stage (Inouye et al. 2002; Kudo and Cooper 2019) and have fewer extreme heat events that reduce the risk of desiccation for large-bodied species (Chown and Gaston 2010; Hamblin et al. 2017). Importantly, *Bombus* spp. share attributes and adaptations, like being large and social, having high intraspecific variation in body size, and are more abundant at higher and cooler elevations, making it difficult to disentangle the relative influence of these attributes, or to tease apart the effects of bumble bee abundance versus climate across sites, on the results. Bumble bees possess unique adaptations to temperate ecosystems that result in locally abundant and widespread species, which can form interactions with many different flower species.

### How do species attributes affect the diet breadth of bumble bee and non-bumble bee species?

As expected, local abundance, phenophase duration, and commonness had a positive association with diet breadth for all species, but only local abundance and phenophase duration had a stronger positive association with diet breadth for bumble bee species compared to non-bumble bee species, suggesting that bumble bees have greater diet flexibility and benefit from sharing activity periods with more resources across time and space than co-occurring non-bumble bee species (Resasco et al. 2021; Vazquez et al. 2009). Flower communities within this ecosystem have been shown to turnover more rapidly across spatial and temporal gradients than bee communities, which requires bumble bees to contend with changing resources across the growing season and different environments (Simanonok and Burkle 2014; Sponsler et al. 2022b). Due to the high rate of floral turnover, bumble bee species have activity periods that overlap with more flower species and potentially increase their diet breadth by forming unique interactions with more flower species over the duration of the growing season. Bumble bee species may also be less constrained to forage from specific plant species that provide resources required for larval development and that have a limited activity period during the growing season (Ogilvie and Forrest 2017). Large local populations of bumble bee species, therefore, increased the chances of sampling bumble bee individuals from different co-occurring flower species. The strong association between patterns of abundance and diet breadth for generalist bee species is consistent with generalist and exotic species in plant–frugivore networks (Coux et al. 2021; Peralta et al. 2020a), indicating that broad diets result from chance interactions and the flexibility to interact with unique partners. Within ecosystems where flower communities turnover rapidly, generalist bee species that persist across space and time connect groups of flower species with different activity periods and have broad diets (Burkle et al. 2021; Resasco et al. 2021).

Contrary to our hypothesis, bee species with higher intraspecific variation in body size did not interact with more flower species, and these relationships may only become apparent at the individual or colony levels in model organisms or experimental settings (Peat et al. 2005). We aggregated interactions from local communities across a naturally heterogeneous landscape, which potentially emphasized the importance of species attributes related to abundance on diet breadth. For instance, bumble bee species may only visit a limited number of flower species within small communities at a local scale, but due to the rapid turnover of floral communities, accumulate interactions with different plant species across sites, resulting in a broad diet breadth for species that occur in many local communities. It could also be the case that the measurement of morphological features of flowers are required to understand trait matching patterns between bees and flowers as a mechanism contributing to bee diet breadth (Burkle et al. 2019; Cariveau et al 2016; Kendall et al. 2019; Klumpers et al. 2019; Stang et al. 2009). Morphological attributes of bee species on diet breadth may be scale-dependent and remain an inconsistent predictor of bee–flower interactions (Bartomeus et al. 2018).

### What is the relationship between bumble bee abundance and the network-level interspecific and intraspecific variation in body size?

The proportion of individuals from a network that were bumble bees had no association with the interspecific, but a positive relationship with the intraspecific variation in body size across networks, underscoring potential associations between bee community composition and the functional diversity of networks. Bumble bee species had higher levels of intraspecific variation in body size compared to non-bumble bee species, which likely led to higher levels of intraspecific trait variation at the network level as the proportion of bumble bee individuals increased across networks. Contrary to our hypothesis, there was no relationship between the proportion of bumble bee individuals in a network and network-level interspecific variation in body size, which was unexpected because increasing the abundance of individuals with similar trait values can cause trait values at the community-level to converge (Classen et al. 2017; Grime 2006; HilleRisLambers et al. 2012). This finding could indicate that there is still interspecific trait variation between species within the *Bombus* genus. Bumble bee species may also replace members of communities as the species with a mean body size that is close to the community-wide mean body size. Despite a historical bias towards interspecific trait variation, intraspecific variation in body size at the community level may be necessary to explain the structure of bee–flower interactions in local communities.

### How does the average diet breadth of bee species vary with interspecific and intraspecific variation in body size?

There was a weak positive association between network-level intraspecific variation in body size and diet breadth, suggesting bumble bee abundance has an indirect association with network generalization by increasing intraspecific trait variation at the community-level. While intraspecific variation in body size did not influence bumble bee mean rarefied interaction richness at the species level, the relationship between intraspecific variation in body size and diet breadth may only emerge at the community level, which considers the effect of co-occurring species on interaction patterns (Violle et al. 2012). For instance, bumble bee species within bumble bee rich communities could face increased pressure to reduce intraspecific or intraguild competition through diet expansion (Bolnick et al. 2011; Fontaine et al. 2008). It is important to note that our study cannot disentangle the correlation between the environment and bumble bee abundance on diet breadth. Regardless, local communities with higher bumble bee abundance likely reflect ecological contexts with fewer constraints where bumble bees are able to exploit a wider range of resources. Environmental variables like elevation, or mean annual temperature may also influence the diet breadth of bee species and have both direct and indirect effects on the diet breadth of bee communities. Future studies that disentangle the correlation between the environment and bumble bee abundance will contribute to a better understanding of the drivers of bee diet breadth.

We demonstrate that among common species within bee–flower networks, characteristics of abundance across space and time have a stronger association with the diet breadth than morphological characteristics for generalist species. Generalists that are abundant within local communities across space and time, and that have the flexibility to interact with more resources, potentially participate in more interactions and form the stable core of ecological networks.

### Supplementary Information

Below is the link to the electronic supplementary material.

Supplementary file1 (DOCX 21 KB)

Supplementary file2 (DOCX 19 KB)

Supplementary file3 (DOCX 20 KB)

Supplementary file4 (XLSX 55 KB)

Supplementary file5 (DOCX 72 KB)

## Data Availability

The datasets generated and/or analyzed during the current study are available from the corresponding author on reasonable request.

## Code availability

The code generated to analyze the data for the current study are available from the corresponding author on reasonable request.
