# Supplementary material for: Bumble bee diet breadth increases with local abundance and phenophase duration, not intraspecific variation in body size
Source: Oecologia. 2024 May 25;205(1):149–62. doi: 10.1007/s00442-024-05560-9 (PMC11144151; doi:10.1007/s00442-024-05560-9)
Supplement: Supplementary file 1 — Supplementary file1 (DOCX 21 KB) [file 442_2024_5560_MOESM1_ESM.docx]

**Supplemental Table 1** Description of locations for sampling bee-flower interactions across southwest Montana. The mean annual precipitation and temperature were derived for each site using worldclim (2.5 minute resolution) and were averaged across all sites within a location. The elevation is reported as a mean and standard deviation of all sites within a location.

| **Location** | **Description** | **Elevation, sd (m)** | **Annual precipitation (mm)** | **Mean annual temperature (C)** | **Sampling Time (min)** | **Rarefied sampling coverage 2018** | **Rarefied sampling coverage 2019** | **Beginning and end dates, 2018** | **Beginning and end dates, 2019** | **Number of sites** | **Distance (least, greatest, median) (m)** | **Network dimensions (bee, flowers, interactions)** | **BB’s (%)** |
| --- | --- | --- | --- | --- | --- | --- | --- | --- | --- | --- | --- | --- | --- |
| Northern Big Belts | Ponderosa pine forest | 1327, 139 | 349 | 16.1 | 7818 | 0.994 | 0.994 | 05/03/2018;  08/07/2019 | 05/13/2019;  08/13/2019 | 17 | 27, 19215, 7801 | 164, 78, 1919 | 8.7 |
| Southern Big Belts | Post-burn, lodgepole pine | 1627, 64 | 431 | 6.2 | 2946 | 0.972 | 0.947 | 05/16/2018;  08/08/2019 | 05/15/2019;  08/12/2019 | 6 | 71, 4925, 1075 | 115, 52, 825 | 10.1 |
| Tenderfoot EF | Montane grassland | 2222, 87 | 595 | -7.7 | 4920 | 1.00 | 0.997 | 07/09/2018;  08/02/2018 | 07/01/2019;  08/08/2019 | 24 | 14, 28064, 8935 | 88, 65, 1669 | 61.4 |
| Boulders | Lodgepole pine forest | 1844, 141 | 432 | 1.3 | 2000 | 0.664 | 1 | 05/09/2018;  08/06/2018 | 06/13/2019;  08/01/2019 | 9 | 308.47, 7908, 3859 | 88, 45, 600 | 48 |
| Elkhorn | Sagebrush Steppe | 1630, 137 | 400 | 7.9 | 1226 | 1 | 1 | 06/07/2018;  07/04/2018 | 05/15/2019;  07/19/2019 | 3 | 2209, 4681, 2543 | 58, 28, 234 | 9.4 |
| Big hole | Sagebrush-forest ecotone | 19618, 58 | 353 | -4.7 | 13640 | 0.927 | 0.995 | 06/14/2018;  07/26/2018 | 06/04/2019;  08/01/2019 | 12 | 365, 19344, 3811 | 150, 67, 1547 | 30 |
