# Supplementary material for: Bumble bee diet breadth increases with local abundance and phenophase duration, not intraspecific variation in body size
Source: Oecologia. 2024 May 25;205(1):149–62. doi: 10.1007/s00442-024-05560-9 (PMC11144151; doi:10.1007/s00442-024-05560-9)
Supplement: Supplementary file 2 — Supplementary file2 (DOCX 19 KB) [file 442_2024_5560_MOESM2_ESM.docx]

**Supplemental Table 2** List of focal plant species used for site selection in locations surrounding Helena.

| **Forb and Shrub Species** | **Common Name** |
| --- | --- |
| *Antennaria microphylla* | Small flowered pussytoes |
| *Antennaria rosea* | Rosy pussytoes |
| *Achillea millefolium* | Yarrow |
| *Penstemon procerus* | Small flowered penstemon |
| *Penstemon attenuatus/albertinus* | Taper leaf penstemon |
| *Eriogonum umbellatum* | Sulphur buckwheat |
| *Lupinus sericeus* | Silky lupine |
| *Anaphalis margaritacea* | Pearly everlasting |
| *Erigeron speciosus* | Showy fleabane |
| *Phacelia hastata* | Silverleaf scorpion weed |
| *Gaillardia aristata* | Blanket flower |
| *Hedysarum boreale* | Boreal sweetvetch |
| *Spiraea betulifolia* | White spirea |
| *Rosa woodsii* | Wood’s rose |
| *Symphoricarpos albus* | Snowberry |
| *Artcostaphylos uva-ursi* | Kinnickinik berry |
| *Berberis repens* | Oregon grape |
| *Eurybia conspicua* | Showy aster |
| *Senecio triangularis* | Arrowleaf groundsel |
| *Cornus sericea* | Red osier dogwood |
| *Salix bebbiana* | Bebb’s willow |
| *Symphyotrichum foliaceum* | Smooth aster |
| *Solidago canadensis* | Canada goldenrod |
