# Supplementary material for: Bumble bee diet breadth increases with local abundance and phenophase duration, not intraspecific variation in body size
Source: Oecologia. 2024 May 25;205(1):149–62. doi: 10.1007/s00442-024-05560-9 (PMC11144151; doi:10.1007/s00442-024-05560-9)
Supplement: Supplementary file 3 — Supplementary file3 (DOCX 20 KB) [file 442_2024_5560_MOESM3_ESM.docx]

**Supplemental Table 3** A description of how the covariates used within this study were measured and standardized. The analysis they are used for pertains to the species-level (rarefaction) or community-level (network) analysis.

| **Covariate** | **Analysis** | **Measurement** | **Standardized** |
| --- | --- | --- | --- |
| Mean rarefied interaction richness | Species-level | Number of unique interactions for a species within a rarefaction run, averaged across runs. | Raw metric |
| *Bombus* | Species-level | Is the species in the *Bombus* genus? | Raw metric |
| Mean rarefied floral richness | Species-level | The number of co-occurring flower species for each individual drawn during a rarefaction run, averaged across all individuals of a species, and then runs. | Raw metric |
| Mean rarefied floral abundance | Species-level | The total inflorescences for each individual drawn during a rarefaction run, averaged across all individuals of a species, and then runs. | Raw metric |
| Mean rarefied CV | Species-level | Coefficient of variation of the intertegular span for a species within a rarefaction run, averaged across runs. | Coefficient of variation |
| Mean rarefied local abundance | Species-level | The number of individuals caught per site in a rarefaction run, averaged across runs | Proportion of sites in a rarefaction run |
| Mean rarefied commonness | Species-level | Number of sites where a species was captured in a rarefaction run, averaged across runs | Proportion of total number of sites in a rarefaction run |
| Mean rarefied phenophase duration | Species-level | Difference in days between the earliest and latest day an individual was caught in a rarefaction run, averaged across runs | Proportion of the range of days sampled in a rarefied network |
| Diet breadth | Community-level | d’ averaged across all species in a network | Relative to a null-model, and against all other species in a network |
| Relative bumble bee abundance | Community-level | Total number of individuals caught within the *Bombus* genus in a network | Proportion of total number of individuals caught within a network. |
| Interspecific variation | Community-level | Difference in mean value of ITD for a species compared to the average value of a community | Z-score transformed community weighted means (Leps et al. 2007) |
| Intraspecific variation | Community-level | Variance in ITD of individuals within a species, summed across all species. | Z-score transformed community weighted means (Leps et al. 2007) |
