# Supplementary material for: Bumble bee diet breadth increases with local abundance and phenophase duration, not intraspecific variation in body size
Source: Oecologia. 2024 May 25;205(1):149–62. doi: 10.1007/s00442-024-05560-9 (PMC11144151; doi:10.1007/s00442-024-05560-9)
Supplement: Supplementary file 5 — Supplementary file5 (DOCX 72 KB) [file 442_2024_5560_MOESM5_ESM.docx]

**Supplemental Table 5** The abundance of each bee species caught from networks. Bee species are identified as bumble bees (‘*Bombus*’) or non-bumble bees (*‘*non-*bombus*’).

| **Species ID** | **Is Bombus?** | **2018 Northern Big Belts** | **2019 Northern Big Belts** | **2018 Boulders** | **2019 Boulders** | **2019 Southern Big Belts** | **2018 Tenderfoot Experimental Forest** | **2019 Tenderfoot Experimental Forest** | **2018 Big Hole** | **2019 Big Hole** | **2018 Elkhorns** | **2019 Elkhorns** | **2018 Southern Big Belts** |
| --- | --- | --- | --- | --- | --- | --- | --- | --- | --- | --- | --- | --- | --- |
| *Andrena illinoiensis* | not.bombus | 23 | 6 | 1 | 0 | 0 | 0 | 0 | 0 | 0 | 0 | 0 | 1 |
| *Andrena frigida* | not.bombus | 3 | 6 | 0 | 0 | 0 | 0 | 0 | 0 | 0 | 0 | 4 | 1 |
| *Lasioglossum ruidosense* | not.bombus | 6 | 8 | 0 | 1 | 2 | 1 | 1 | 4 | 28 | 0 | 0 | 3 |
| *Andrena cressonii* | not.bombus | 20 | 19 | 0 | 0 | 0 | 0 | 0 | 0 | 0 | 0 | 0 | 0 |
| *Lasioglossum inconditum* | not.bombus | 43 | 22 | 0 | 0 | 5 | 1 | 0 | 0 | 0 | 6 | 4 | 3 |
| *Andrena vierecki* | not.bombus | 16 | 3 | 0 | 0 | 0 | 0 | 0 | 0 | 0 | 0 | 0 | 0 |
| *Lasioglossum aff.caducum* | not.bombus | 7 | 2 | 0 | 0 | 1 | 0 | 0 | 0 | 0 | 0 | 0 | 1 |
| *Lasioglossum cooleyi* | not.bombus | 8 | 9 | 0 | 0 | 1 | 1 | 0 | 0 | 0 | 0 | 8 | 2 |
| *Andrena striatifrons* | not.bombus | 2 | 2 | 0 | 0 | 0 | 0 | 0 | 0 | 0 | 0 | 0 | 0 |
| *Halictus rubicundus* | not.bombus | 8 | 17 | 0 | 1 | 7 | 3 | 16 | 19 | 18 | 0 | 4 | 20 |
| *Halictus confusus* | not.bombus | 7 | 13 | 0 | 0 | 7 | 0 | 0 | 1 | 6 | 0 | 0 | 16 |
| *Lasioglossum laevissimum* | not.bombus | 5 | 1 | 0 | 3 | 5 | 0 | 1 | 1 | 0 | 0 | 0 | 8 |
| *Lasioglossum nevadense* | not.bombus | 4 | 0 | 0 | 0 | 0 | 0 | 0 | 0 | 0 | 0 | 1 | 0 |
| *Osmia lignaria propinqua* | not.bombus | 4 | 16 | 0 | 0 | 7 | 0 | 0 | 0 | 0 | 0 | 0 | 2 |
| *Andrena merriami* | not.bombus | 1 | 0 | 0 | 0 | 0 | 0 | 0 | 0 | 7 | 0 | 0 | 0 |
| *Lasioglossum* sp.f5 | not.bombus | 1 | 0 | 0 | 0 | 0 | 0 | 0 | 0 | 0 | 0 | 0 | 0 |
| *Andrena transnigra* | not.bombus | 4 | 5 | 0 | 1 | 1 | 0 | 0 | 3 | 18 | 2 | 1 | 0 |
| *Andrena topazana* | not.bombus | 1 | 5 | 1 | 5 | 3 | 1 | 5 | 1 | 5 | 0 | 0 | 1 |
| *Andrena sola* | not.bombus | 15 | 25 | 0 | 0 | 0 | 0 | 0 | 0 | 0 | 2 | 6 | 0 |
| *Anthophora pacifica* | not.bombus | 1 | 19 | 0 | 0 | 1 | 0 | 0 | 0 | 0 | 4 | 0 | 1 |
| *Andrena subtilis* | not.bombus | 2 | 2 | 0 | 0 | 0 | 0 | 0 | 0 | 0 | 0 | 0 | 0 |
| *Andrena miranda* | not.bombus | 33 | 6 | 0 | 1 | 10 | 1 | 5 | 0 | 2 | 2 | 1 | 4 |
| *Andrena crataegi* | not.bombus | 35 | 63 | 0 | 0 | 5 | 0 | 0 | 0 | 0 | 0 | 18 | 21 |
| *Andrena milwaukeensis* | not.bombus | 2 | 9 | 0 | 1 | 1 | 0 | 1 | 4 | 18 | 1 | 0 | 0 |
| *Andrena medionitens* | not.bombus | 1 | 14 | 0 | 0 | 2 | 0 | 0 | 2 | 0 | 0 | 0 | 1 |
| *Andrena carlini* | not.bombus | 1 | 1 | 0 | 0 | 0 | 0 | 0 | 1 | 2 | 0 | 0 | 0 |
| *Lasioglossum lineatulum* | not.bombus | 3 | 2 | 0 | 0 | 0 | 0 | 0 | 0 | 0 | 0 | 0 | 0 |
| *Panurginus torchoi* | not.bombus | 9 | 1 | 0 | 0 | 0 | 0 | 0 | 0 | 5 | 0 | 0 | 3 |
| *Andrena nigrocaerulea* | not.bombus | 4 | 16 | 0 | 1 | 1 | 1 | 0 | 5 | 7 | 1 | 0 | 1 |
| *Andrena miserabilis* | not.bombus | 2 | 0 | 0 | 1 | 0 | 0 | 0 | 0 | 0 | 0 | 0 | 0 |
| *Andrena persimulata* | not.bombus | 2 | 6 | 0 | 3 | 0 | 0 | 0 | 0 | 0 | 1 | 5 | 0 |
| *Andrena sigmundi* | not.bombus | 1 | 9 | 0 | 0 | 2 | 0 | 3 | 0 | 0 | 0 | 3 | 0 |
| *Halictus tripartitus* | not.bombus | 12 | 25 | 0 | 1 | 0 | 0 | 0 | 0 | 0 | 0 | 0 | 1 |
| *Lasioglossum albipenne* | not.bombus | 20 | 45 | 0 | 0 | 3 | 0 | 0 | 0 | 0 | 7 | 5 | 9 |
| *Lasioglossum tenax* | not.bombus | 1 | 2 | 0 | 0 | 1 | 0 | 0 | 0 | 0 | 0 | 0 | 1 |
| *Andrena cupreotincta* | not.bombus | 2 | 14 | 0 | 0 | 0 | 0 | 0 | 0 | 0 | 0 | 1 | 1 |
| *Andrena angustitarsata* | not.bombus | 0 | 4 | 0 | 0 | 0 | 0 | 0 | 0 | 0 | 0 | 1 | 0 |
| *Lasioglossum sisymbrii* | not.bombus | 1 | 4 | 0 | 0 | 1 | 0 | 0 | 0 | 1 | 1 | 0 | 2 |
| *Anthophora ursina* | not.bombus | 15 | 9 | 0 | 0 | 1 | 0 | 1 | 3 | 5 | 4 | 5 | 0 |
| *Andrena torchio* | not.bombus | 0 | 3 | 0 | 0 | 9 | 0 | 1 | 0 | 0 | 0 | 0 | 0 |
| *Andrena auricoma* | not.bombus | 0 | 3 | 0 | 0 | 0 | 0 | 0 | 0 | 0 | 0 | 2 | 0 |
| *Lasioglossum obnubilum* | not.bombus | 0 | 4 | 0 | 3 | 0 | 0 | 0 | 2 | 0 | 0 | 1 | 0 |
| *Andrena hippotes* | not.bombus | 0 | 12 | 0 | 0 | 0 | 0 | 0 | 0 | 0 | 0 | 1 | 0 |
| *Andrena candida* | not.bombus | 0 | 2 | 0 | 0 | 0 | 0 | 0 | 0 | 0 | 0 | 2 | 0 |
| *Hoplitis albifrons.argentifrons* | not.bombus | 6 | 14 | 0 | 3 | 12 | 11 | 9 | 1 | 8 | 0 | 0 | 1 |
| *Hylaeus episcopalis* | not.bombus | 0 | 1 | 0 | 0 | 2 | 0 | 0 | 0 | 0 | 0 | 0 | 6 |
| *Hoplitis fulgida.fulgida* | not.bombus | 2 | 20 | 8 | 4 | 13 | 15 | 19 | 14 | 23 | 0 | 1 | 5 |
| *Andrena atriceps* | not.bombus | 0 | 15 | 0 | 0 | 0 | 1 | 7 | 0 | 0 | 0 | 0 | 0 |
| *Osmia tersula* | not.bombus | 0 | 3 | 0 | 1 | 0 | 3 | 2 | 0 | 8 | 0 | 0 | 1 |
| *Lasioglossum incompletum* | not.bombus | 0 | 2 | 0 | 0 | 0 | 0 | 0 | 0 | 0 | 0 | 0 | 0 |
| *Lasioglossum anhypops* | not.bombus | 0 | 1 | 0 | 5 | 2 | 0 | 0 | 3 | 3 | 0 | 0 | 1 |
| *Andrena nivalis* | not.bombus | 3 | 10 | 0 | 0 | 18 | 1 | 0 | 0 | 0 | 0 | 1 | 2 |
| *Lasioglossum egregium* | not.bombus | 0 | 3 | 0 | 2 | 0 | 0 | 0 | 1 | 2 | 1 | 0 | 0 |
| *Osmia kincaidii* | not.bombus | 2 | 36 | 0 | 0 | 1 | 0 | 0 | 0 | 0 | 0 | 0 | 1 |
| *Andrena prunorum* | not.bombus | 2 | 19 | 0 | 0 | 2 | 0 | 0 | 0 | 0 | 0 | 0 | 0 |
| *Hoplitis producta* | not.bombus | 4 | 5 | 0 | 0 | 2 | 0 | 0 | 6 | 10 | 0 | 0 | 11 |
| *Hoplitis hypocrita* | not.bombus | 1 | 2 | 0 | 0 | 0 | 0 | 0 | 0 | 0 | 0 | 0 | 0 |
| *Osmia densa* | not.bombus | 23 | 15 | 0 | 0 | 5 | 1 | 0 | 0 | 0 | 0 | 0 | 14 |
| *Bombus centralis* | bombus | 31 | 7 | 3 | 3 | 15 | 6 | 1 | 3 | 8 | 1 | 2 | 1 |
| *Dufourea trochantera* | not.bombus | 13 | 35 | 0 | 0 | 0 | 0 | 0 | 0 | 0 | 0 | 0 | 0 |
| *Colletes consors* | not.bombus | 2 | 32 | 0 | 0 | 1 | 1 | 0 | 0 | 4 | 0 | 1 | 0 |
| *Eucera edwardsii* | not.bombus | 1 | 8 | 0 | 0 | 0 | 0 | 0 | 0 | 0 | 21 | 9 | 1 |
| *Calliopsis personata* | not.bombus | 0 | 1 | 0 | 0 | 0 | 0 | 0 | 0 | 0 | 0 | 0 | 0 |
| *Ceratina neomexicana* | not.bombus | 7 | 20 | 0 | 0 | 5 | 0 | 0 | 0 | 0 | 0 | 2 | 2 |
| *Osmia atrocyanea* | not.bombus | 9 | 15 | 0 | 0 | 3 | 0 | 0 | 2 | 1 | 0 | 2 | 0 |
| *Hoplitis grinnelli* | not.bombus | 0 | 6 | 0 | 0 | 0 | 0 | 0 | 0 | 1 | 0 | 0 | 0 |
| *Osmia cyanella* | not.bombus | 0 | 3 | 0 | 0 | 2 | 0 | 0 | 0 | 0 | 0 | 0 | 0 |
| *Osmia tristella* | not.bombus | 1 | 5 | 2 | 5 | 1 | 22 | 48 | 9 | 9 | 1 | 0 | 1 |
| *Osmia coloradensis* | not.bombus | 9 | 6 | 0 | 17 | 2 | 5 | 7 | 27 | 47 | 0 | 1 | 9 |
| *Osmia pusilla* | not.bombus | 11 | 10 | 3 | 7 | 4 | 2 | 5 | 24 | 28 | 0 | 1 | 2 |
| *Hylaeus mesillae/rudbeckiae* | not.bombus | 8 | 5 | 0 | 1 | 0 | 0 | 0 | 0 | 5 | 0 | 0 | 1 |
| *Osmia paradisica* | not.bombus | 0 | 1 | 1 | 11 | 1 | 11 | 104 | 19 | 46 | 1 | 1 | 0 |
| *Osmia montana montana* | not.bombus | 12 | 8 | 4 | 4 | 1 | 4 | 1 | 5 | 20 | 0 | 0 | 1 |
| *Bombus bifarius* | bombus | 57 | 15 | 78 | 74 | 38 | 108 | 165 | 141 | 203 | 1 | 0 | 9 |
| *Bombus mixtus* | bombus | 3 | 0 | 31 | 38 | 0 | 216 | 96 | 21 | 11 | 0 | 0 | 0 |
| *Bombus flavifrons* | bombus | 0 | 0 | 10 | 17 | 3 | 43 | 53 | 2 | 1 | 1 | 1 | 1 |
| *Megachile gemula* | not.bombus | 0 | 2 | 3 | 0 | 2 | 2 | 4 | 0 | 2 | 0 | 0 | 2 |
| *Bombus melanopygus* | bombus | 0 | 0 | 3 | 6 | 0 | 138 | 109 | 4 | 2 | 0 | 0 | 0 |
| *Megachile melanophaea* | not.bombus | 7 | 2 | 4 | 11 | 0 | 9 | 23 | 7 | 5 | 0 | 0 | 1 |
| *Bombus frigidus* | bombus | 0 | 0 | 2 | 0 | 0 | 1 | 0 | 5 | 6 | 0 | 0 | 0 |
| *Anthidium tenuiflorae* | not.bombus | 0 | 0 | 1 | 0 | 1 | 0 | 0 | 0 | 1 | 0 | 0 | 0 |
| *Anthophora terminalis* | not.bombus | 2 | 10 | 5 | 1 | 5 | 0 | 0 | 1 | 1 | 0 | 0 | 2 |
| *Megachile pugnata* | not.bombus | 2 | 3 | 2 | 0 | 10 | 1 | 0 | 0 | 0 | 0 | 0 | 6 |
| *Coelioxys modesta* | not.bombus | 0 | 0 | 1 | 1 | 0 | 0 | 0 | 0 | 0 | 0 | 0 | 0 |
| *Heriades cressoni* | not.bombus | 66 | 11 | 2 | 0 | 0 | 0 | 0 | 0 | 0 | 0 | 0 | 8 |
| *Hylaeus modestus* | not.bombus | 0 | 0 | 5 | 2 | 10 | 13 | 0 | 7 | 3 | 0 | 0 | 5 |
| *Osmia bucephala* | not.bombus | 0 | 1 | 0 | 7 | 0 | 0 | 0 | 1 | 4 | 0 | 0 | 0 |
| *Lasioglossum* sp1.sky | not.bombus | 0 | 0 | 0 | 1 | 0 | 0 | 0 | 0 | 0 | 0 | 0 | 0 |
| *Hoplitis robusta* | not.bombus | 1 | 0 | 2 | 3 | 0 | 3 | 2 | 8 | 12 | 0 | 0 | 0 |
| *Dufourea maura* | not.bombus | 8 | 1 | 0 | 1 | 1 | 6 | 14 | 0 | 4 | 0 | 0 | 9 |
| *Hylaeus annulatus* | not.bombus | 0 | 0 | 7 | 0 | 0 | 10 | 5 | 0 | 2 | 0 | 0 | 2 |
| *Lasioglossum ovaliceps* | not.bombus | 1 | 1 | 1 | 0 | 0 | 0 | 0 | 0 | 0 | 0 | 0 | 0 |
| *Hylaeus modestus citrinifrons* | not.bombus | 0 | 0 | 2 | 0 | 1 | 0 | 1 | 0 | 0 | 0 | 0 | 0 |
| *Hylaeus wootoni* | not.bombus | 7 | 4 | 20 | 8 | 4 | 0 | 0 | 0 | 3 | 0 | 0 | 18 |
| *Osmia subaustralis* | not.bombus | 1 | 0 | 1 | 3 | 0 | 12 | 10 | 2 | 14 | 0 | 0 | 4 |
| *Hylaeus coloradensis* | not.bombus | 0 | 0 | 0 | 2 | 1 | 0 | 0 | 7 | 0 | 0 | 0 | 0 |
| *Osmia juxta* | not.bombus | 11 | 13 | 0 | 3 | 29 | 0 | 1 | 1 | 0 | 0 | 0 | 7 |
| *Bombus occidentalis* | bombus | 0 | 0 | 0 | 2 | 4 | 4 | 14 | 4 | 2 | 0 | 0 | 0 |
| *Hoplitis spoliata* | not.bombus | 0 | 0 | 0 | 3 | 1 | 0 | 1 | 0 | 0 | 0 | 0 | 0 |
| *Stelis montana* | not.bombus | 1 | 0 | 0 | 2 | 1 | 3 | 3 | 3 | 2 | 0 | 0 | 1 |
| *Ashmeadiella californica* | not.bombus | 5 | 7 | 3 | 1 | 0 | 3 | 6 | 0 | 6 | 0 | 0 | 1 |
| *Megachile angelarum* | not.bombus | 0 | 0 | 0 | 1 | 0 | 0 | 0 | 0 | 0 | 0 | 0 | 0 |
| *Osmia trevoris* | not.bombus | 1 | 6 | 1 | 0 | 0 | 1 | 2 | 6 | 12 | 0 | 1 | 0 |
| *Hylaeus basalis* | not.bombus | 0 | 2 | 0 | 1 | 7 | 0 | 2 | 0 | 0 | 0 | 0 | 4 |
| *Ashmeadiella pronitens* | not.bombus | 0 | 0 | 0 | 1 | 0 | 0 | 1 | 0 | 1 | 0 | 0 | 0 |
| *Osmia aff.enixa* | not.bombus | 0 | 0 | 0 | 6 | 0 | 0 | 3 | 0 | 0 | 0 | 0 | 0 |
| *Andrena vicinoides* | not.bombus | 0 | 13 | 0 | 1 | 12 | 0 | 0 | 0 | 0 | 0 | 0 | 1 |
| *Osmia inermis* | not.bombus | 0 | 0 | 0 | 0 | 3 | 0 | 1 | 0 | 0 | 0 | 0 | 1 |
| *Andrena knuthiana* | not.bombus | 0 | 0 | 0 | 1 | 20 | 0 | 0 | 0 | 0 | 0 | 0 | 0 |
| *Lasioglossum sedi* | not.bombus | 1 | 6 | 0 | 0 | 8 | 8 | 3 | 7 | 27 | 1 | 8 | 0 |
| *Lasioglossum nigroviridae* | not.bombus | 0 | 0 | 0 | 1 | 46 | 0 | 0 | 1 | 0 | 0 | 0 | 16 |
| *Megachile perihirta* | not.bombus | 4 | 4 | 0 | 0 | 2 | 0 | 9 | 3 | 2 | 0 | 0 | 2 |
| *Osmia aff.paradisica* | not.bombus | 0 | 0 | 1 | 0 | 0 | 6 | 1 | 20 | 7 | 0 | 0 | 0 |
| *Panurginus atriceps* | not.bombus | 0 | 6 | 0 | 0 | 0 | 18 | 5 | 3 | 0 | 0 | 0 | 15 |
| *Bombus cent.flav* | bombus | 1 | 0 | 0 | 0 | 0 | 1 | 2 | 0 | 0 | 0 | 0 | 0 |
| *Halictus ligatus* | not.bombus | 10 | 19 | 0 | 0 | 0 | 0 | 3 | 0 | 0 | 6 | 1 | 2 |
| *Megachile frigida* | not.bombus | 0 | 0 | 2 | 1 | 5 | 1 | 5 | 3 | 1 | 0 | 0 | 2 |
| *Bombus rufocinctus* | bombus | 32 | 7 | 0 | 0 | 10 | 12 | 13 | 3 | 2 | 2 | 0 | 3 |
| *Bombus californicus* | bombus | 0 | 0 | 0 | 2 | 0 | 0 | 1 | 0 | 4 | 5 | 5 | 0 |
| *Hylaeus verticalis* | not.bombus | 0 | 8 | 5 | 2 | 2 | 3 | 1 | 0 | 0 | 0 | 0 | 3 |
| *Osmia phaceliae?* | not.bombus | 5 | 0 | 1 | 0 | 1 | 6 | 2 | 10 | 2 | 0 | 0 | 1 |
| *Andrena specularia* | not.bombus | 0 | 0 | 1 | 0 | 0 | 2 | 0 | 3 | 0 | 0 | 0 | 0 |
| *Osmia pentstemonis* | not.bombus | 3 | 0 | 1 | 1 | 0 | 11 | 11 | 1 | 0 | 0 | 0 | 3 |
| *Ceratina nanula* | not.bombus | 5 | 7 | 1 | 0 | 4 | 0 | 0 | 3 | 6 | 0 | 0 | 0 |
| *Osmia albolateralis* | not.bombus | 17 | 14 | 0 | 1 | 2 | 1 | 1 | 3 | 12 | 0 | 0 | 2 |
| *Osmia marginipennis* | not.bombus | 0 | 32 | 0 | 1 | 0 | 0 | 0 | 1 | 1 | 0 | 0 | 0 |
| *Osmia californica* | not.bombus | 3 | 6 | 0 | 2 | 3 | 0 | 0 | 0 | 0 | 0 | 0 | 0 |
| *Osmia dolerosa* | not.bombus | 0 | 0 | 0 | 1 | 0 | 0 | 0 | 0 | 0 | 0 | 0 | 0 |
| *Lasioglossum perdifficile* | not.bombus | 2 | 19 | 0 | 0 | 0 | 0 | 0 | 0 | 0 | 1 | 0 | 0 |
| *Andrena lawrencei* | not.bombus | 6 | 6 | 0 | 0 | 0 | 0 | 0 | 0 | 0 | 0 | 0 | 1 |
| *Andrena amphibola* | not.bombus | 2 | 8 | 0 | 0 | 1 | 1 | 0 | 2 | 0 | 2 | 2 | 0 |
| *Lasioglossum ebmerellum* | not.bombus | 4 | 2 | 0 | 0 | 0 | 0 | 0 | 0 | 0 | 0 | 3 | 0 |
| *Lasioglossum trizonatum* | not.bombus | 1 | 2 | 0 | 1 | 0 | 0 | 1 | 8 | 11 | 0 | 0 | 1 |
| *Agapostemon virescens* | not.bombus | 2 | 5 | 0 | 0 | 0 | 0 | 0 | 0 | 0 | 0 | 1 | 0 |
| *Andrena melanochroa* | not.bombus | 2 | 2 | 0 | 0 | 2 | 0 | 0 | 0 | 3 | 0 | 0 | 5 |
| *Andrena salicifloris* | not.bombus | 4 | 1 | 0 | 2 | 0 | 2 | 19 | 3 | 20 | 0 | 0 | 0 |
| *Andrena vicina* | not.bombus | 1 | 5 | 0 | 1 | 1 | 0 | 0 | 1 | 0 | 0 | 0 | 0 |
| *Agapostemon texanus angelicus* | not.bombus | 3 | 2 | 0 | 0 | 0 | 0 | 1 | 0 | 0 | 12 | 4 | 2 |
| *Osmia brevis* | not.bombus | 9 | 0 | 0 | 0 | 5 | 0 | 17 | 3 | 6 | 0 | 0 | 0 |
| *Andrena petristis* | not.bombus | 1 | 0 | 0 | 0 | 0 | 0 | 0 | 0 | 0 | 0 | 0 | 0 |
| *Andrena thaspii* | not.bombus | 1 | 2 | 0 | 0 | 0 | 0 | 1 | 0 | 8 | 0 | 1 | 1 |
| *Bombus nevadensis* | bombus | 1 | 0 | 0 | 0 | 0 | 0 | 0 | 0 | 0 | 0 | 0 | 0 |
| *Heriades carinata* | not.bombus | 15 | 3 | 0 | 0 | 0 | 0 | 0 | 0 | 0 | 0 | 0 | 7 |
| *Heriades variolosa* | not.bombus | 5 | 1 | 0 | 0 | 0 | 0 | 0 | 0 | 0 | 0 | 0 | 0 |
| *Bombus huntii* | bombus | 7 | 1 | 0 | 0 | 0 | 0 | 0 | 0 | 0 | 0 | 0 | 0 |
| *Andrena sladeni* | not.bombus | 1 | 14 | 0 | 0 | 0 | 0 | 1 | 0 | 0 | 0 | 0 | 0 |
| *Melecta pacifica* | not.bombus | 4 | 1 | 0 | 0 | 0 | 0 | 0 | 0 | 0 | 0 | 1 | 0 |
| *Osmia ednae* | not.bombus | 1 | 1 | 0 | 0 | 2 | 0 | 0 | 0 | 0 | 0 | 0 | 10 |
| *Megachile relativa* | not.bombus | 0 | 1 | 0 | 0 | 1 | 9 | 3 | 1 | 0 | 0 | 0 | 0 |
| *Andrena scurra* | not.bombus | 0 | 2 | 0 | 0 | 0 | 0 | 0 | 0 | 0 | 0 | 0 | 0 |
| *Anthophora bomboides* | not.bombus | 0 | 1 | 0 | 0 | 0 | 0 | 0 | 0 | 0 | 0 | 0 | 0 |
| *Colletes fulgidus* | not.bombus | 3 | 4 | 0 | 0 | 2 | 0 | 0 | 0 | 0 | 0 | 0 | 1 |
| *Ashmeadiella bucconis* | not.bombus | 0 | 1 | 0 | 0 | 0 | 0 | 0 | 0 | 0 | 0 | 0 | 0 |
| *Lasioglossum* sp3.sky | not.bombus | 0 | 1 | 0 | 0 | 2 | 0 | 0 | 0 | 0 | 0 | 0 | 0 |
| *Lasioglossum* wg.sp1 | not.bombus | 0 | 0 | 0 | 0 | 0 | 0 | 0 | 9 | 45 | 0 | 0 | 0 |
| *Andrena evoluta* | not.bombus | 0 | 0 | 0 | 0 | 0 | 0 | 0 | 5 | 13 | 0 | 0 | 0 |
| *Bombus sylvicola* | bombus | 1 | 0 | 0 | 0 | 0 | 6 | 8 | 4 | 32 | 2 | 0 | 0 |
| *Lasioglossum* wg.sp10 | not.bombus | 0 | 0 | 0 | 0 | 0 | 0 | 0 | 2 | 0 | 0 | 0 | 0 |
| *Lasioglossum* f14 | not.bombus | 0 | 0 | 0 | 0 | 0 | 0 | 0 | 5 | 0 | 0 | 0 | 0 |
| *Lasioglossum* wg.sp3 | not.bombus | 0 | 0 | 0 | 0 | 0 | 0 | 0 | 0 | 12 | 0 | 0 | 0 |
| *Andrena nigrihirta* | not.bombus | 0 | 0 | 0 | 0 | 0 | 0 | 0 | 0 | 2 | 0 | 0 | 0 |
| *Lasioglossum* sp.f1 | not.bombus | 0 | 0 | 0 | 0 | 0 | 0 | 0 | 0 | 18 | 0 | 0 | 0 |
| *Lasioglossum* wg.sp2 | not.bombus | 0 | 0 | 0 | 0 | 0 | 0 | 0 | 0 | 2 | 0 | 0 | 0 |
| *Osmia longula* | not.bombus | 0 | 1 | 0 | 0 | 0 | 0 | 0 | 0 | 4 | 1 | 0 | 0 |
| *Andrena commoda* | not.bombus | 0 | 0 | 0 | 0 | 0 | 0 | 0 | 0 | 0 | 1 | 0 | 0 |
| *Bombus fervidus* | bombus | 0 | 1 | 0 | 0 | 0 | 0 | 1 | 1 | 3 | 2 | 0 | 0 |
| *Osmia grindeliae* | not.bombus | 0 | 0 | 0 | 0 | 1 | 0 | 1 | 0 | 0 | 0 | 1 | 0 |
| *Lasioglossum abundipunctum* | not.bombus | 1 | 0 | 0 | 0 | 3 | 0 | 0 | 0 | 2 | 1 | 7 | 0 |
| *Lasioglossum pruinosum* | not.bombus | 0 | 1 | 0 | 0 | 0 | 2 | 0 | 0 | 0 | 2 | 3 | 0 |
| *Lasioglossum succinipenne* | not.bombus | 3 | 1 | 0 | 0 | 0 | 0 | 0 | 0 | 0 | 0 | 4 | 0 |
| *Colletes kincaidii* | not.bombus | 0 | 0 | 0 | 0 | 0 | 0 | 0 | 0 | 1 | 0 | 1 | 0 |
| *Megachile fidelis* | not.bombus | 5 | 1 | 0 | 0 | 0 | 0 | 0 | 0 | 0 | 0 | 0 | 0 |
| *Megachile apicalis* | not.bombus | 13 | 2 | 0 | 0 | 0 | 0 | 0 | 0 | 0 | 0 | 0 | 0 |
| *Lasioglossum* sp2.sky | not.bombus | 0 | 0 | 0 | 0 | 0 | 1 | 0 | 0 | 0 | 0 | 0 | 0 |
| *Osmia bruneri* | not.bombus | 7 | 0 | 0 | 0 | 0 | 0 | 0 | 0 | 10 | 0 | 0 | 3 |
| *Megachile montivaga* | not.bombus | 1 | 0 | 0 | 0 | 0 | 0 | 0 | 0 | 2 | 0 | 0 | 1 |
| *Anthidium utahense* | not.bombus | 1 | 0 | 0 | 0 | 0 | 0 | 0 | 0 | 0 | 0 | 0 | 3 |
| *Andrena trevoris* | not.bombus | 3 | 2 | 0 | 0 | 10 | 0 | 0 | 0 | 0 | 0 | 0 | 12 |
| *Ashmeadiella cactorum* | not.bombus | 1 | 0 | 0 | 0 | 1 | 0 | 0 | 0 | 1 | 0 | 0 | 0 |
| *Lasioglossum marinense* | not.bombus | 14 | 0 | 0 | 2 | 9 | 1 | 0 | 0 | 1 | 0 | 0 | 6 |
| *Megachile rotundata* | not.bombus | 2 | 0 | 0 | 0 | 0 | 0 | 0 | 0 | 0 | 0 | 0 | 0 |
| *Dianthidium subparvum* | not.bombus | 3 | 0 | 0 | 0 | 0 | 0 | 0 | 1 | 1 | 0 | 0 | 0 |
| *Melissodes confusa* | not.bombus | 1 | 0 | 0 | 0 | 0 | 0 | 0 | 0 | 0 | 0 | 0 | 0 |
| *Lasioglossum liliputense* | not.bombus | 0 | 3 | 0 | 0 | 0 | 0 | 0 | 0 | 0 | 0 | 0 | 1 |
| *Lasioglossum punctatoventre* | not.bombus | 0 | 1 | 0 | 0 | 0 | 0 | 0 | 0 | 0 | 0 | 0 | 1 |
| *Panurginus ineptus* | not.bombus | 0 | 1 | 0 | 0 | 1 | 2 | 1 | 0 | 0 | 0 | 0 | 4 |
| *Hylaeus mesillae* | not.bombus | 1 | 1 | 0 | 0 | 0 | 0 | 0 | 0 | 0 | 0 | 0 | 0 |
| *Melissodes microsticta* | not.bombus | 2 | 0 | 0 | 0 | 0 | 0 | 0 | 1 | 0 | 0 | 0 | 2 |
| *Osmia iridis* | not.bombus | 0 | 2 | 0 | 0 | 0 | 0 | 0 | 0 | 0 | 0 | 0 | 0 |
| *Stelis monticola* | not.bombus | 0 | 1 | 0 | 0 | 0 | 0 | 0 | 1 | 0 | 0 | 0 | 0 |
| *Megachile wheeleri* | not.bombus | 0 | 1 | 0 | 0 | 0 | 0 | 0 | 0 | 0 | 0 | 0 | 0 |
| *Osmia sedula* | not.bombus | 1 | 0 | 0 | 0 | 0 | 0 | 0 | 0 | 0 | 0 | 0 | 0 |
| *Anthidium mormonum* | not.bombus | 3 | 0 | 0 | 0 | 3 | 0 | 0 | 0 | 1 | 0 | 0 | 0 |
| *Osmia nigrifrons* | not.bombus | 1 | 0 | 0 | 0 | 0 | 0 | 0 | 0 | 1 | 0 | 0 | 0 |
| *Andrena walleyi* | not.bombus | 0 | 0 | 0 | 0 | 0 | 0 | 0 | 1 | 5 | 0 | 0 | 0 |
| *Andrena* sp. | not.bombus | 0 | 0 | 0 | 0 | 0 | 0 | 0 | 4 | 0 | 0 | 0 | 0 |
| *Lasioglossum* wg.sp4 | not.bombus | 0 | 0 | 0 | 0 | 0 | 0 | 0 | 0 | 6 | 0 | 0 | 0 |
| *Colletes nigrifrons* | not.bombus | 0 | 0 | 0 | 0 | 0 | 0 | 0 | 8 | 25 | 0 | 0 | 0 |
| *Melissodes* sp.f3 | not.bombus | 0 | 0 | 0 | 0 | 0 | 0 | 0 | 0 | 1 | 0 | 0 | 0 |
| *Andrena* spp. | not.bombus | 0 | 0 | 0 | 0 | 0 | 0 | 0 | 0 | 2 | 0 | 0 | 0 |
| *Hylaeus rudbeckiae* | not.bombus | 1 | 0 | 0 | 0 | 0 | 0 | 0 | 0 | 0 | 0 | 0 | 0 |
| *Bombus appositus* | bombus | 3 | 0 | 0 | 0 | 1 | 2 | 0 | 0 | 1 | 0 | 0 | 5 |
| *Osmia pikei* | not.bombus | 0 | 0 | 0 | 0 | 1 | 0 | 1 | 0 | 0 | 0 | 0 | 0 |
| *Lasioglossum* ephialtum? | not.bombus | 0 | 0 | 0 | 1 | 1 | 0 | 1 | 0 | 0 | 0 | 0 | 0 |
| *Coelioxys porterae* | not.bombus | 0 | 0 | 0 | 0 | 1 | 0 | 1 | 0 | 0 | 0 | 0 | 0 |
| *Melissodes desponsus* | not.bombus | 0 | 0 | 0 | 0 | 0 | 0 | 1 | 0 | 0 | 0 | 0 | 0 |
| *Andrena bradleyi* | not.bombus | 0 | 1 | 0 | 0 | 0 | 0 | 0 | 0 | 0 | 0 | 0 | 0 |
| *Dianthidium ulkei* | not.bombus | 0 | 1 | 0 | 0 | 0 | 0 | 0 | 0 | 0 | 0 | 0 | 0 |
| *Hoplitis truncata* | not.bombus | 0 | 2 | 0 | 0 | 2 | 0 | 0 | 0 | 0 | 0 | 0 | 0 |
| *Triepeolus epeolus sp.* | not.bombus | 0 | 0 | 0 | 0 | 0 | 0 | 1 | 0 | 0 | 0 | 0 | 0 |
| *Lasioglossum occidentale* | not.bombus | 0 | 0 | 0 | 0 | 0 | 0 | 0 | 1 | 0 | 0 | 0 | 0 |
| *Lasioglossum* wg.sp7 | not.bombus | 0 | 0 | 0 | 0 | 0 | 0 | 0 | 3 | 7 | 0 | 0 | 0 |
| *Stelis* bh.f1 | not.bombus | 0 | 0 | 0 | 0 | 0 | 0 | 0 | 2 | 1 | 0 | 0 | 0 |
| *Nomada* sp.f1.sky | not.bombus | 0 | 0 | 0 | 0 | 0 | 0 | 0 | 0 | 6 | 0 | 0 | 0 |
| *Osmia* ?proxima | not.bombus | 0 | 0 | 0 | 0 | 0 | 0 | 0 | 0 | 3 | 0 | 0 | 0 |
| *Nomada* sp.f9.sky | not.bombus | 0 | 0 | 0 | 0 | 0 | 0 | 0 | 1 | 2 | 0 | 0 | 0 |
| *Osmia physariae* | not.bombus | 0 | 0 | 0 | 0 | 0 | 0 | 0 | 0 | 5 | 0 | 0 | 0 |
| *Osmia aff.nigrifrons* | not.bombus | 0 | 0 | 0 | 0 | 0 | 2 | 0 | 0 | 0 | 0 | 0 | 0 |
| *Lasioglossum* sp.2 | not.bombus | 0 | 0 | 0 | 0 | 0 | 1 | 0 | 0 | 0 | 0 | 0 | 0 |
| *Halictus brunneiventre* | not.bombus | 0 | 0 | 0 | 0 | 0 | 0 | 0 | 1 | 0 | 0 | 0 | 0 |
| *Lasioglossum brunneiventre* | not.bombus | 0 | 0 | 0 | 0 | 0 | 0 | 0 | 3 | 0 | 0 | 0 | 0 |
| *Hylaeus* wgsp1 | not.bombus | 0 | 0 | 0 | 0 | 0 | 0 | 0 | 6 | 0 | 0 | 0 | 0 |
| *Lasioglossum* wg.sp8 | not.bombus | 0 | 0 | 0 | 0 | 0 | 0 | 0 | 1 | 1 | 0 | 0 | 0 |
| *Nomada* sp.f12.sky | not.bombus | 0 | 0 | 0 | 0 | 0 | 0 | 0 | 0 | 1 | 0 | 0 | 0 |
| *Lasioglossum lilliputense* | not.bombus | 0 | 0 | 0 | 0 | 0 | 0 | 0 | 0 | 6 | 0 | 0 | 0 |
| *Lasioglossum nr.dialictus* | not.bombus | 0 | 0 | 0 | 0 | 0 | 0 | 0 | 4 | 0 | 0 | 0 | 0 |
| *Stelis calliphorina* | not.bombus | 0 | 0 | 0 | 0 | 0 | 0 | 0 | 2 | 0 | 0 | 0 | 0 |
| *Lasioglossum semicaerulean* | not.bombus | 0 | 0 | 0 | 0 | 0 | 0 | 0 | 1 | 0 | 0 | 0 | 0 |
| *Anthophora urbana* | not.bombus | 0 | 0 | 0 | 0 | 0 | 0 | 0 | 2 | 0 | 0 | 0 | 0 |
| *Nomada* sp.f3.sky | not.bombus | 0 | 0 | 0 | 0 | 0 | 0 | 0 | 0 | 3 | 0 | 0 | 0 |
| *Sphecodes* sp.f3 | not.bombus | 0 | 0 | 0 | 0 | 0 | 0 | 0 | 0 | 2 | 0 | 0 | 0 |
| *Andrena berberidis* | not.bombus | 0 | 0 | 0 | 0 | 0 | 0 | 0 | 0 | 1 | 0 | 0 | 0 |
| *Panurginus atrifrons* | not.bombus | 0 | 0 | 0 | 0 | 0 | 0 | 0 | 0 | 8 | 0 | 0 | 0 |
| *Sphecodes* sp.f1 | not.bombus | 0 | 0 | 0 | 0 | 0 | 0 | 0 | 0 | 1 | 0 | 0 | 0 |
| *Stelis* bh.f2 | not.bombus | 0 | 0 | 0 | 0 | 0 | 0 | 0 | 0 | 1 | 0 | 0 | 0 |
| *Hoplitis* f.sp1 | not.bombus | 0 | 0 | 0 | 0 | 0 | 0 | 0 | 0 | 1 | 0 | 0 | 0 |
| *Osmia* odontagaster.sp2 | not.bombus | 0 | 0 | 0 | 0 | 0 | 0 | 1 | 0 | 0 | 0 | 0 | 0 |
| *Lasioglossum cressonii* | not.bombus | 0 | 0 | 0 | 1 | 0 | 0 | 0 | 0 | 0 | 0 | 0 | 0 |
| *Andrena laminibucca* | not.bombus | 0 | 0 | 0 | 4 | 0 | 0 | 0 | 0 | 0 | 0 | 0 | 0 |
| *Andrena nasonii* | not.bombus | 0 | 0 | 0 | 1 | 0 | 0 | 0 | 0 | 0 | 0 | 0 | 0 |
| *Andrena pallidifovea* | not.bombus | 0 | 0 | 0 | 0 | 0 | 0 | 0 | 0 | 2 | 0 | 0 | 0 |
| *Nomada* sp.f4.sky | not.bombus | 0 | 0 | 0 | 0 | 0 | 0 | 0 | 0 | 1 | 0 | 0 | 0 |
| *Nomada* sp.f6 | not.bombus | 0 | 0 | 0 | 0 | 0 | 0 | 0 | 0 | 2 | 0 | 0 | 0 |
| *Hylaeus* affinis? | not.bombus | 0 | 0 | 0 | 0 | 0 | 0 | 0 | 0 | 2 | 0 | 0 | 0 |
| *Andrenidae* sp1 | not.bombus | 0 | 0 | 0 | 0 | 0 | 0 | 0 | 1 | 0 | 0 | 0 | 0 |
| *Lasioglossum aff.tenax* | not.bombus | 0 | 0 | 0 | 0 | 0 | 0 | 0 | 1 | 0 | 0 | 0 | 0 |
| *Andrena saccata* | not.bombus | 0 | 0 | 0 | 0 | 2 | 0 | 0 | 0 | 1 | 0 | 0 | 0 |
| *Andrena astragali* | not.bombus | 0 | 0 | 0 | 0 | 0 | 0 | 0 | 0 | 1 | 0 | 0 | 0 |
| *Lasioglossum* sp.f2 | not.bombus | 0 | 0 | 0 | 0 | 0 | 0 | 0 | 0 | 2 | 0 | 0 | 0 |
| *Lasioglossum* wg.sp6 | not.bombus | 0 | 0 | 0 | 0 | 0 | 0 | 0 | 0 | 1 | 0 | 0 | 0 |
| *Osmia* sp1 | not.bombus | 0 | 0 | 0 | 0 | 0 | 0 | 0 | 2 | 0 | 0 | 0 | 0 |
| *Nomada* sp.f13 | not.bombus | 0 | 0 | 0 | 0 | 0 | 0 | 0 | 0 | 1 | 0 | 0 | 0 |
| *Nomada* sp.f11.sky | not.bombus | 0 | 0 | 0 | 0 | 0 | 0 | 0 | 0 | 1 | 0 | 0 | 0 |
| *Lasioglossum* wg.sp9 | not.bombus | 0 | 0 | 0 | 0 | 0 | 0 | 0 | 1 | 0 | 0 | 0 | 0 |
| *Lasioglossum* wg.sp5 | not.bombus | 0 | 0 | 0 | 0 | 0 | 0 | 0 | 0 | 1 | 0 | 0 | 0 |
| *Triepelous paenepectoralis* | not.bombus | 0 | 0 | 0 | 0 | 0 | 0 | 0 | 0 | 0 | 0 | 0 | 1 |
| *Megachile lapponica* | not.bombus | 0 | 0 | 0 | 0 | 0 | 0 | 0 | 0 | 0 | 0 | 0 | 1 |
| *Dufourea holocyanea* | not.bombus | 0 | 0 | 0 | 0 | 3 | 0 | 0 | 0 | 0 | 0 | 0 | 0 |
| *Lasioglossum knerei* | not.bombus | 0 | 0 | 0 | 0 | 1 | 0 | 0 | 0 | 0 | 0 | 0 | 0 |
| *Lasioglossum titusi* | not.bombus | 0 | 0 | 0 | 0 | 2 | 0 | 0 | 0 | 0 | 0 | 0 | 0 |
| *Coelioxys alternata* | not.bombus | 0 | 0 | 0 | 0 | 1 | 0 | 0 | 0 | 0 | 0 | 0 | 0 |
| *Specodes* sp.f1 | not.bombus | 0 | 0 | 0 | 0 | 0 | 0 | 0 | 1 | 0 | 0 | 0 | 0 |
| *Sphecodes* sp.f4 | not.bombus | 0 | 0 | 0 | 0 | 0 | 0 | 0 | 0 | 1 | 0 | 0 | 0 |
| *Ashmeadiella meliloti* | not.bombus | 0 | 0 | 0 | 0 | 0 | 0 | 0 | 0 | 1 | 0 | 0 | 0 |
| *Bombus griseocollis* | bombus | 0 | 0 | 0 | 0 | 0 | 0 | 0 | 0 | 1 | 0 | 0 | 0 |
| *Anthidium clypeodontatum* | not.bombus | 0 | 0 | 0 | 0 | 0 | 0 | 0 | 0 | 1 | 0 | 0 | 0 |
